# Disrupted dispersal and its genetic consequences: Comparing protected and threatened baboon populations (*Papio papio*) in West Africa

**DOI:** 10.1371/journal.pone.0194189

**Published:** 2018-04-03

**Authors:** Maria Joana Ferreira da Silva, Gisela H. Kopp, Catarina Casanova, Raquel Godinho, Tânia Minhós, Rui Sá, Dietmar Zinner, Michael W. Bruford

**Affiliations:** 1 Organisms and Environment Division, School of Biosciences, Cardiff University, Cardiff, Wales, United Kingdom; 2 CIBIO/InBio, Centro de Investigação em Biodiversidade e Recursos Genéticos, Universidade do Porto, Campus Agrário de Vairão, Vairão, Portugal; 3 CAPP, School of Social and Political Sciences, University of Lisbon, Rua Almerindo Lessa, Lisboa, Portugal; 4 Cognitive Ethology Laboratory, German Primate Center, Göttingen, Germany; 5 Departamento de Biologia, Faculdade de Ciências da Universidade do Porto, Rua do Campo Alegre, Porto, Portugal; 6 Department of Zoology, Faculty of Sciences, University of Johannesburg, Auckland Park, South Africa; 7 Departamento de Antropologia, Faculdade de Ciências Sociais e Humanas, Universidade Nova de Lisboa, Lisboa, Portugal; 8 Centre for Research in Anthropology (CRIA), Instituto Universitário de Lisboa, Lisboa, Portugal; 9 IGC, Instituto Gulbenkian de Ciência, Rua da Quinta Grande, Oeiras, Portugal; 10 Departamento de Ciências Ambientais, Universidade Lusófona da Guiné, Rua Vitorino Costa, Bissau, Guiné-Bissau; 11 Research Centre for Anthropology and Health, Universidade de Coimbra, Calçada Martim de Freitas, Coimbra, Portugal; 12 Sustainable Places Research Institute, Cardiff University, Cardiff, Wales, United Kingdom; Australian National University, AUSTRALIA

## Abstract

Dispersal is a demographic process that can potentially counterbalance the negative impacts of anthropogenic habitat fragmentation. However, mechanisms of dispersal may become modified in populations living in human-dominated habitats. Here, we investigated dispersal in Guinea baboons (*Papio papio*) in areas with contrasting levels of anthropogenic fragmentation, as a case study. Using molecular data, we compared the direction and extent of sex-biased gene flow in two baboon populations: from Guinea-Bissau (GB, fragmented distribution, human-dominated habitat) and Senegal (SEN, continuous distribution, protected area). Individual-based Bayesian clustering, spatial autocorrelation, assignment tests and migrant identification suggested female-mediated gene flow at a large spatial scale for GB with evidence of contact between genetically differentiated males at one locality, which could be interpreted as male-mediated gene flow in southern GB. Gene flow was also found to be female-biased in SEN for a smaller scale. However, in the southwest coastal part of GB, at the same geographic scale as SEN, no sex-biased dispersal was detected and a modest or recent restriction in GB female dispersal seems to have occurred. This population-specific variation in dispersal is attributed to behavioural responses to human activity in GB. Our study highlights the importance of considering the genetic consequences of disrupted dispersal patterns as an additional impact of anthropogenic habitat fragmentation and is potentially relevant to the conservation of many species inhabiting human-dominated environments.

## Introduction

Dispersal is usually sex-biased in mammals and birds, *i*.*e*., one sex disperses more frequently and/or further while the opposite sex remains and reproduces in its natal area [1–2]. This sex-biased dispersal pattern is usually consistent within and between closely related species [3–4]. However, local changes in ecological and demographic factors (*e*.*g*. intrinsic patch features such as food availability, size and isolation, population density, intra- and inter-specific interactions, sex ratio, relatedness) can alter an individual’s dispersal strategy [2, 5–7]. Individuals that otherwise would remain philopatric may disperse to seek reproductive opportunities or because the costs of dispersal surpass the benefits, individuals may therefore reproduce in their natal areas [4–5]. As a consequence, the dispersal pattern of the population, usually biased towards one sex, may become less obvious or reversed [2, 4, 8].

Since dispersal promotes genetic exchange between populations, changes in dispersal patterns are expected to influence a population’s genetic composition and conservation requirements [7, 9]. If gene flow increases, differentiated sub-populations may come into contact and exchange genes in contact zones, leading to potential genetic “swamping” and loss of local adaptations [8]. On the other hand, if gene flow is constrained, populations may become more structured, leading to a loss of genetic variation through drift and possibly a reduction of reproductive fitness if mating options become limited to kin resulting in inbreeding [8, 10].

Human activities may drive changes in population demography and connectivity, which can modify population dispersal patterns [2, 5]. Changes in sex-biased dispersal have been described for several taxa in populations inhabiting human-altered environments, including primates (*e*.*g*. *Pan troglodytes verus*, [11], *Macaca cyclopis*, [12], *Ateles belzebuth*, [13]). Moreover, female dispersal in Old World primates is frequently limited by the degree of overlap of a group’s home range with others [14]. In human-dominated environments, low population density and isolation of groups across the landscape may reduce the extent of overlap between home ranges, increasing the cost of dispersal for females. As a consequence, females may not disperse from their natal group, which in turn may increase genetic differentiation between sub-populations and lead to a less pronounced female-biased dispersal pattern [2, 8].

However, predicting the influence of human activities on dispersal is not straightforward. Its effects may depend on an individuals’ ability to move between populations and to reproduce, on the intensity of anthropogenic disturbance and on the species’ ability to cope with such disruption [15–16], which is often difficult to measure. Additionally, human activities may have contrasting effects on gene flow (*e*.*g*. decreasing, [17]; increasing, [18]) and on group composition [19–21]. As a result, these effects can easily be overlooked or mistaken for the characteristic pattern of a species, particularly when local human practices are illegal and concealed and when only one population is studied. Identifying how and whether anthropogenic activities influence dispersal may thus require comparison among populations subject to different levels of human disturbance [7, 9].

Here, we investigated population differences in dispersal behaviour in Guinea baboons (*Papio papio*). Our study was focused on two geographically distinct populations—southern Guinea-Bissau and Senegal—that inhabit environments with contrasting levels of human disturbance. Baboons from southern Guinea-Bissau inhabit a human-altered environment and have been extensively hunted, mainly for meat consumption [22–24]. The species’ distribution in Guinea-Bissau is highly fragmented, with few groups outside national parks [22, 25]. In contrast, the Niokolo Koba National Park population in Senegal, the geographic scale of which is comparable to the Guinea-Bissau study site, is not significantly affected by human activity and is continuously distributed within the park limits [26, 27].

Dispersal in Guinea baboons is thought to be female-biased. Previous studies have inferred long-term female-mediated gene flow across the species distribution [28], including the Senegalese [29] and the Guinea-Bissau population [23, 25], and on-going female-biased dispersal in Senegal [29]. Sex-biased dispersal in the last few generations has not been studied for the Guinea-Bissau population. However, results from our previous work suggested that the intensification of human activities might have modified the Guinea-Bissau population structure in the last thirty years (3–4 generations, at 12–15 years [30]). As the population in southern Guinea-Bissau is very fragmented, we initially hypothesized that populations inhabiting protected areas would be isolated, but evidence for on-going gene flow included: i) differentiated genetic units were found to be in contact, in particular in one protected area, where the location of genetic discontinuities and anthropogenic landscape features do not seem to be correlated, and ii) significant genetic similarity was found between samples distanced 115.5 km apart, with higher genetic dissimilarity found at 66 km corresponding to an area impacted by anthropogenic activities and low group density [25]. Thus, genetic structure within the Guinea-Bissau population suggested a recent increase in dispersal distances [25], an unexpected result for a species with female-biased dispersal and which may signal a recent change in sex-biased dispersal in the population.

Our aim in this study was to follow up our previous work by comparing patterns of sex-biased gene flow (direction and magnitude) at similar geographic scales between Guinea-Bissau and a less disturbed environment (in Senegal). By applying molecular tools to study sex-biased dispersal we can detect differences starting just a few generations ago and test its impact on population structure [31, 32]. While the philopatric sex is expected to show higher natal social group relatedness, display greater genetic differentiation among groups and thus higher population structure, the dispersing sex is expected to homogenize genetic differentiation across groups and thus display more similar allele frequencies across subpopulations [31, 32]. Given the higher degree of human disturbance across Guinea-Bissau when compared to Senegal, we expected to find differences in sex-specific population genetic structure between the two populations, which would indicate differences in the sex-biased dispersal. More specifically, we expected to find no difference in the degree of genetic structure between males and females in Guinea-Bissau (*e*.*g*. no sex-biased dispersal) and a higher degree of genetic structure in males when compared with females in Senegal (*e*.*g*. female-biased dispersal).

## Methods

### Study areas

**Fig 1:**
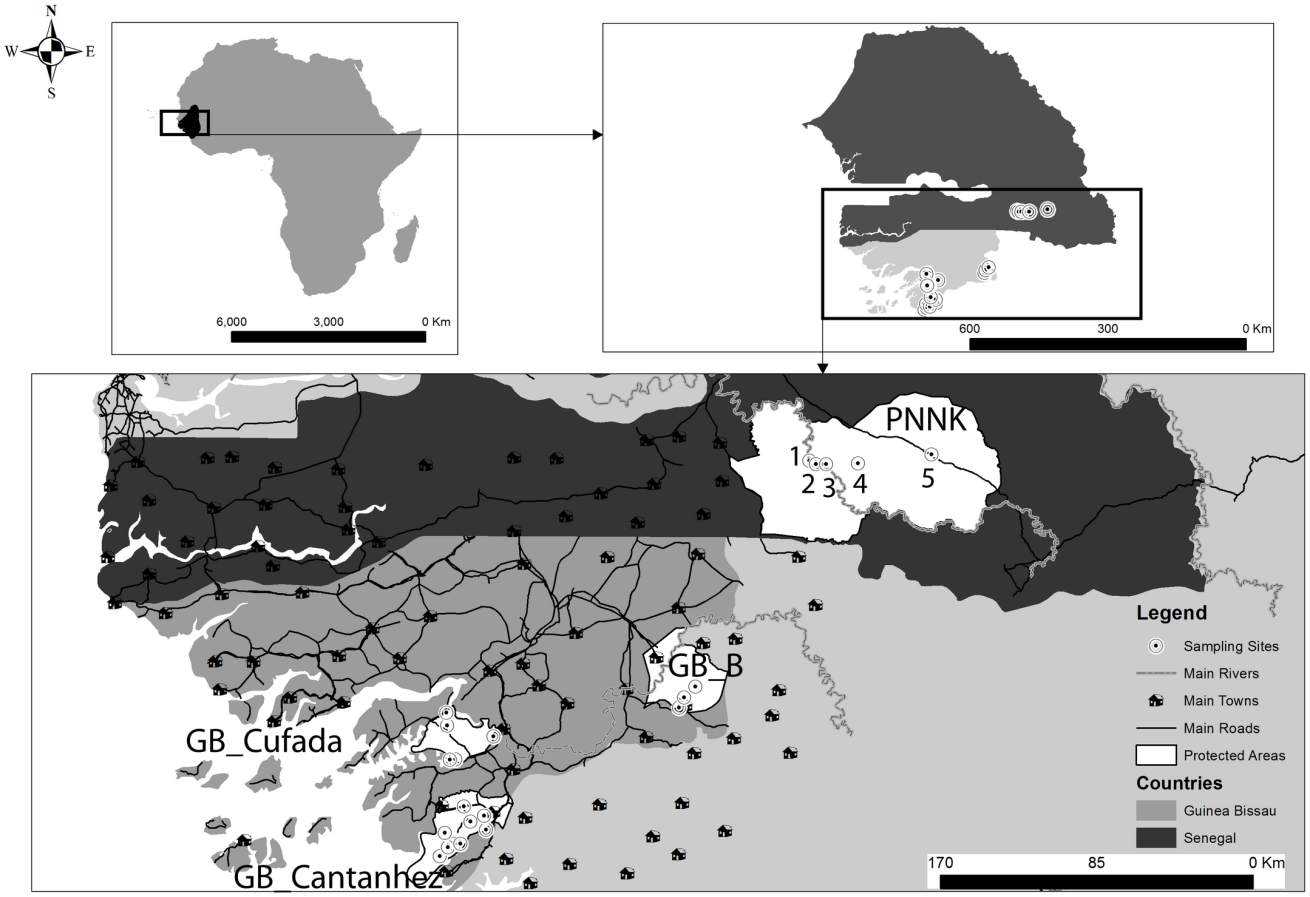
Study areas. Top left: Black shading in overview map indicates the distribution of Guinea baboons (adapted from IUCN). Top right: Guinea-Bissau (GB—country area in light grey) and Senegal (SEN—country area in dark grey). Sampled sites indicated by black-dotted white circles. Bottom: Most important anthropogenic features (main roads, villages and towns) and sampling sites in Senegal (Niokolo Koba National Park—PNNK, sampling sites: 1—Gue Damantan, 2—Simenti, 3—Camp Lion, 4—Lingue Kountou, and 5—Niokolo) and in Guinea-Bissau (GB_B—Boé Natural Park, GB_Cufada—Cufada Lagoons Natural Park, and GB_Cantanhez—Cantanhez Woodlands Natural Park). The two main rivers (Corubal in Guinea-Bissau and the Gambia River in Senegal) are indicated by a grey line. The comparison of dispersal patterns between Senegal and Guinea-Bissau was done for the geographic scale of 66 km (the spatial scale between sampling sites in Senegal and between GB_Cantanhez and GB_Cufada in GB).

The study encompasses two Guinea baboon populations—Guinea-Bissau (GB) and Senegal (SEN), separated by approximately 150 km (Fig 1). Previous analyses using samples collected across the entire distribution of the species found that GB and SEN constitute differentiated genetic clusters [33–34].

In GB, the study area includes three geographically distinct localities—1) Cantanhez Woodlands National Park (GB_Cantanhez, north limit 11.389835° -14.810265° to south limit 11.010386° -15.234415°), 2) Cufada Lagoons Natural Park (GB_Cufada, north limit: 11.753801° -15.185281° to south limit: 11.627405° -14.903575°), and 3) Boé Natural Park (GB_Boé, north limit 12.206995° -14.005278° to south limit 11.825659° to -13.863576°). GB_Cantanhez and GB_Cufada are located in the southwest coastal part of GB and are separated roughly by 50 km. GB_Cantanhez (total area 106 767 ha) is located on a peninsula connected to mainland GB by a 25–30 km isthmus. GB_Cufada (a total area of 89,000 ha) is surrounded by the Corubal River in the north and Buba channel in the south and delimitated by two main towns—Buba and Fulacunda—in the southwest and in the northeast limits, respectively. GB_Boé is located in the southeast of the country and is separated from GB_Cantanhez and GB_Cufada roughly by 120 Km and 100 km, respectively. GB_Boé is delimitated in the west by the Corubal River and in the east by the Fefine River. In SEN, the study was carried out within the Niokolo Koba National Park (PNNK, north limit: 13.329490° -13.442568° and south limit: 12.644166° -13.094790°, a total area of 913,000 ha), across a maximum distance of 65 km between sampling sites.

The area between the GB’s Cantanhez and Cufada parks, which is comparable to the spatial scale among sampling sites in PNNK, has a higher degree of human disturbance (Fig 1). IBAP (*Instituto da Biodiversidade e Áreas Protegidas*, Institute for Biodiversity and Protected Areas), the national authority responsible for the management of protected areas in GB, has a set of rules in place that limit hunting practices and deforestation to a certain extent within the limits of protected areas. A considerable extension of the original habitat has been converted to crop areas outside protected areas in GB, with a variety of human infrastructure, including paved roads connecting the south of the country to the capital city and medium-size towns (Fig 1). Groups of baboons are rarely observed outside protected areas in GB [22]. Likewise, the populations of Guinea baboons outside protected areas in Senegal are thought to have suffered a widespread decline in the last thirty years [35–36] and fragmentation of the distribution is likely. To the best of our knowledge, the populations between GB_Boé and PNNK have not been recently surveyed.

Permission to work in the Guinea-Bissau protected areas (*e*.*g*. Cantanhez, Cufada, and Boé) was issued by IBAP. Permission to work in PNNK was issued by Direction des Parcs Nationaux and Ministère de l'Environnement et de la Protection de la Nature de la Republique du Senegal (Attestation 0383/24/03/2009 and 0373/10/3/2012).

### Sampling

We reanalysed a microsatellite *loci* (STR) dataset generated by previous studies composed of 143 unique multi-locus genotypes (54 males and 89 females) sampled in GB’s protected areas (GB_Cantanhez N = 71, GB_Cufada N = 51, and GB_Boé N = 21 genotypes) [25, 37] and 165 unique genotypes (97 males and 68 females) sampled within PNNK area in SEN [29] (Fig 1; Table 1; Table A in S1 Appendix). Genotypes were obtained non-invasively from faecal samples, collected randomly from unhabituated and unidentified individuals in baboon home ranges, in foraging paths and sleeping sites (for details on sampling techniques, see [25, 29, 34]). During sample collection, the location of each sample was recorded using a GPS receiver.

**Table 1:** Inference of dispersal patterns at multiple geographic scales for Guinea-Bissau and Senegal.

Scale^a^	Sub-set of samples^b^	Analyses	Predictions	Results	Dispersal pattern inferred
**165**	GB165 N = 143 54 M and 89 F Includes GB_Cantanhez, GB_Cufada, and GB_Boé	STRUCTURE	Higher population structure for the philopatric sex.	K = 2 No significant differences between the sexes. M and F at Cufada assigned to cluster 1 (41.2% M and 77% F) and cluster 2 (18% M and 12% F)	No sex bias in dispersal Both sexes disperse to Cufada
		Global spatial autocorrelation	Correlograms of M and F distinct if sex bias in dispersal Genetic structure of philopatric sex significantly positive at shorter distances and negative at larger distances.	M significantly more similar than F at shorter distances and more dissimilar at larger distances. Negative spatial autocorrelation between GB_Cantanhez and GB_Cufada for both sexes. Significant positive autocorrelation between GB_Boé and GB_Cufada for F.	Female-biased
		GENECLASS	Dispersing individuals display a high probability of being born at other locations other than where sampled	Six first generation migrants (M and F) identified in all locations	Both sexes disperse
		Assignment index N = 81 without missing data. GB_Cantanhez: 26 F and 14 M GB_Cufada: 28 F and 13 M	The dispersing sex is expected to display lower and negative *mAIc* and higher *vAIc* than the philopatric sex	GB_Cantanhez No significant differences between the sexes. GB_Cufada F *mAIc* > 0 and M *mAIc* < 0 M *vAIc* > F *vAIc*	Majority of Cufada M can be considered immigrants
**66**	GB66 N = 111 37 M and 74 F GB_Cantanhez, GB_Cufada up to 66 km	STRUCTURE	Higher population structure for the philopatric sex.	No genetic structure detected.	No sex-biased dispersal detected
		Global spatial autocorrelation	Correlograms of M and F distinct if sex bias in dispersal Genetic structure of philopatric sex significantly positive at shorter distances and negative at larger distances.	No difference between M and F.	
	SEN66 N = 165 97 M and 68 F PKKN	STRUCTURE N = 158 67 F and 91 M	Higher population structure for the philopatric sex.	Higher genetic structure for M. NK harbours females assigned to both genetic clusters.	Female- biased
		Global spatial autocorrelation	Correlograms of M and F distinct if sex bias in dispersal Genetic structure of philopatric sex significantly positive at shorter distances and negative at larger distances.	Spatial autocorrelation is heterogeneous between sexes	
		Assignment index N = 151 without missing data 90 M and 61 F	The dispersing sex is expected to display lower and negative *mAIc* and higher *vAIc* than the philopatric sex	No difference between M and F.	

^a^ Note that dispersal patterns were inferred at three geographic scales (165 km, 66, and 26 km) but the table shows the 165 and 66 km scales only.

^b^ Sub-sets of samples (“GB165”, “GB66”, and “SEN66”). GB165 included all samples collected in Guinea-Bissau to a maximum of 165 km, the distance between the furthest samples collected in Cantanhez and Boé. A threshold of 66 km was set to perform comparative analyses between SEN and GB (for the criteria used to chose these distances, please see S4 Appendix). GB66 included samples from Cantanhez Woodlands National Park (GB_Cantanhez) and Cufada Lagoons Natural Park (GB_Cufada), distanced to a maximum of 66 km. SEN66 included samples from Niokolo Koba National Park area, distanced to the furthest distance of 65.0 km. Note that results of STRUCTURE for SEN66 shown in the table are for a re-run of STRUCTURE after removing seven samples from the sub-set (see text for details).

In GB, sampling was carried out at 17 sampling sites within protected areas (average number of genotypes per sampling site = 8.4, varying between 3 and 21, the average distance between sampling sites = 64.9 km, ranging from 2.6 to 164.5 km, Figure A and Tables A and B in S1 Appendix). Sampling sites in Guinea-Bissau are unlikely to represent independent social groups. In Senegal, social parties may divide into smaller groups or merge into larger ones several times a day [38] and we assumed a similar social organization in Guinea-Bissau. Samples collected in GB at one site may represent groups of individuals of the same social unit (*i*.*e*., the individuals were foraging or sleeping together before defecating) but neighbouring sampling sites might not be independent (*i*.*e*., the groups might have slipped before or may have merged after the sampling moment).

In SEN, sampling was carried out at five sampling sites in an almost continuous design (average number of genotypes per sampling site = 33, varying between 108 and 11, the average distance between sampling sites = 18.1 km, varying between 4.1 and 65.0 km) (Table C in S1 Appendix). Most samples collected in SEN stem from the Simenti community, a troop under study by the Cognitive Ethology Laboratory of the German Primate Center (DPZ).

### Microsatellite analyses and sex determination

DNA samples were analyzed using thirteen microsatellite *loci* used in common by [25] and [29] (S2 Appendix). Microsatellite *loci* are human-derived and cross-amplify in *Papio* [39–40] following procedures detailed in S2 Appendix. Allelic dropout rate (ADO) and false allele rate (FA) were estimated according to [41] for single PCRs. The probability of identity (pID) and the probability of identity among sibs (pIDsib) [42] were computed.

The sex of individual samples was determined using a molecular protocol detailed in S3 Appendix, which have been previously optimized by [29, 37].

### Statistical analyses

We performed individual-level analyses to avoid making *a priori* assumptions on the grouping pattern of the individuals. Given the large geographic distance separating GB_Cufada, GB_Cantanhez and GB_Boé, those regions were considered as independent geographic distinct subpopulations.

#### Genetic diversity

Allelic Richness (*AR*) per locus, corrected for unequal sample sizes by rarefaction [43], and Linkage Disequilibrium (LD) between all pairs of loci per database (GB and SEN) were estimated using FSTAT v2.9.3.2 [44]. Observed (*H*_*o*_) and expected heterozygosity (*H*_*e*_) per locus and tests for Hardy-Weinberg Equilibrium (HWE) were computed using GenAlEx v6.3 [45]. We estimated the genetic diversity per geographically distinct localities (SEN, GB_Cantanhez, GB_Cufada and GB_Boé) and separately for males and females. GenAlEx was used to estimate the number of alleles (*N*_*a*_), the number of effective alleles (*N*_*e*,_ *i*.*e*., the number of alleles with equal frequencies), *H*_*o*_, *H*_*e*_, Unbiased expected heterozygosity (*UH*_*e*_) and *F*_*is*_.

#### Sex-biased gene flow

Dispersal distances are unknown for the Guinea baboons. In other baboon species, dispersing individuals usually travel less than 20 km between neighbouring groups (*Papio cynocephalus*: 15–22 km [46] or 11–19.1 km [47]) and dispersal may occur multiple times during the life span of individuals. Nevertheless, given that Guinea baboons can travel up to 15 km daily, and that significant genetic differentiation was found at geographic distances larger than 50 km [25, 29], a sex bias in dispersal in the Guinea baboon might be detected at greater distances than for other species (*i*.*e*., > 20 km).

Considering that dispersal is a scale-dependent process [48] and the difference in geographic scale of the study area in the two populations (GB < 165 km and SEN < 65 km), we assembled sub-sets of samples collected at different spatial scales to test for sex bias in dispersal in the human-dominated environment of GB and compare dispersal patterns between GB and SEN.

We started by testing sex-biased dispersal at a broad scale (*i*.*e*., to a maximum linear distance of 165 km) by assembling all genotypes sampled in GB_Cantanhez, GB_Cufada, and GB_Boé in a sub-set hereafter named “GB165” (54 males and 89 females, Table 1; S1 Appendix). Sex-biased dispersal at a broad geographic scale including SEN could not be tested because the genotypes in SEN were sampled at a maximum Euclidean linear distance of 65.0 km (Table C in S1 Appendix) and a sampling gap of approximately 136 km is present between the closest samples of GB_Boe and SEN.

Individual-based clustering implemented in STRUCTURE v2.1 [49] was used to investigate genetic structure. We inferred clusters (K) between one and ten, with five independent replicates, using the admixture model (default parameters) and assuming correlated allele frequencies. We used the sampling location of individuals (*i*.*e*., GB_Cantanhez, GB_Cufada and GB_Boé, Table 1; S1 Appendix) as a prior in the model ([50], *e*.*g*. [29, 34]). The Locprior model is expected to improve the assignment of individuals to putative genetic clusters when the structure is weak, without driving any particular structure if a genetic signal is absent and by overwriting the sampling information when the genetic ancestry of individuals is unrelated with the respective sampling site [29, 50]. Each run was preceded by a burn-in of 100,000 steps followed by MCMC runs of 1,000,000 iterations. The most likely number of clusters (K) in STRUCTURE was estimated using the highest estimated log-likelihood, the *ad hoc* statistic ΔK [51], both retrieved using STRUCTURE HARVESTER v6.8 [52], and the posterior probability of K, calculated according to [49]. We used different criteria to determine the most likely K because the *ad hoc* statistic ΔK is not suitable when a structure is absent (*i*.*e*., K = 1 [51]). We averaged the individual probability of assignment (q) over the five independent runs and used the plot of ranked partial membership of each individual to each cluster to detect the minimum q of the samples clearly assigned to genetic clusters [53, 54]. We calculated the proportion of males and females assigned to each genetic cluster per sampling region.

Spatial autocorrelation, as estimated in GenAlEx 6.3, was used to compare the spatial genetic structure between the sexes [55]. The autocorrelation coefficient *r* (-1 to 1) measures the genetic similarity (*r* > 0) or dissimilarity (*r <* 0) between pairs of individuals grouped in distance classes. Significant spatial structure (P < 0.05) is present when *r* lies outside a 95% upper and lower confidence interval, obtained by permutation with 9,999 replicates [55]. We calculated the pairwise codominant genotypic distance and the linear geographical distances between individuals. Geographical distances were calculated using the GPS coordinates of the samples registered in the UTM (Universal Transversal Mercator) coordinate system and translated to km using GenAIEx. Analyses were performed at the 165 km scale (Table 1; S1 Appendix) and samples were grouped in ten distance classes ([0–17[, [17–34[, [34–51[, [51–68[, [68–85[, [85–102[, [102–119[, [119–136[, and [136–153] km, starting point). The width of the distance classes was chosen among other possible combinations to allow for i) a direct comparison between sub-sets, using the same geographic distances for males and females and including sufficient number of pairs of individuals in each class (*i*.*e*., n _pairwise comparisons_) to perform the analyses, and to ii) likely encompass dispersal distances for the Guinea baboon (see above). Squared paired-sample t-tests (t^2^) and the omega (ω) test criteria were used to compare the heterogeneity between correlograms of males and females for single and overall distance classes [56]. Significant differences of the heterogeneity test between correlograms (P < 0.01, [57]) were tested using 9,999 bootstraps [55]. With sex-biased dispersal, the correlograms of males and females are expected to be distinct and the philopatric sex should display significant positive genetic structure at shorter distances and significant negative genetic structure at longer distances [58]. Analyses were repeated including the GB_Cantanhez, GB_Cufada datasets only (41 males and 81 females) to assess the effect of excluding sampling sites in the spatial autocorrelation pattern. Samples were grouped in five distance classes ([0–16.5[, [16.5–33[, [33–49.5[, [49.5–66[, and [66–82.5[) and analyses were carried out following the described procedures.

To detect first-generation migrants (*i*.*e*., the individuals with a high probability of being born at one sampling locality), we divided the GB165 dataset in GB_Cantanhez, GB_Cufada, and GB_Boé and used GENECLASS version 2.0 [59] to estimate two likelihood-based tests statistics: L_h_ (more adequate when the source population might not have been sampled) and L_h_/L_max_ (better when all source populations were presumably sampled) [60]. A Bayesian method [61] and the Paetkau [62] resampling Monte Carlo algorithm (10,000 simulations and an alpha level of 0.01) were used to identify the critical values distinguishing between residents and migrants. We expected to find a higher number of first-generation migrants of the dispersing sex.

We estimated the assignment index *AIc*, mean corrected assignment index *mAIc* and variance of the assignment index *vAIc* in GenAlEx 6.3. *AIc*, which can be defined as the probability of a genotype assigned to the locality where the individuals were sampled, is expected to be negative for dispersing individuals [63]. If dispersal is sex-biased, the dispersing sex is expected to display a lower than expected frequency of rare alleles in relation to the population where the individual was sampled [63]. Significant differences in *mAIc* and *vAIc* can be found between the dispersing and the philopatric sex [63]. Specifically, the dispersing sex is expected to display lower and negative *mAIc* and higher *vAIc* than the philopatric sex [63]. In GenAlEx, this analysis uses samples with no missing data. Therefore, *AIc*, *mAIc*, and *vAIc* were estimated for 26 females and 14 males sampled at GB_Cantanhez and 28 females and 13 males sampled at GB_Cufada. Since most of the genotypes sampled at Boé had missing data (*e*.*g*. seven out of eight females identified), GB_Boé was excluded from these analyses. Significance was tested using a Mann-Whitney U-test.

To compare sex-biased dispersal between GB and SEN, we estimated the pairwise Euclidean linear geographic distances between samples using GenAlEx 6.3 for the two populations separately (S4 Appendix). Sampling sites in GB165 were selected to be part of the comparative analyses between the two populations using the following criteria: i) distance between sampling sites would include probable dispersal distances for the Guinea baboon (*i*.*e*., > 20 km), ii) selected sampling sites in GB would match inter-site distances in SEN, and iii) selected sampling sites would be comparable in sample size (S4 Appendix). We set the thresholds of 66 km and 26 km (S4 Appendix).

The sub-sets of samples up to a maximum Euclidean distance of 66 km are hereafter named as “GB66” and “SEN66” (Table 1). These subsets are used to compare sex-mediated gene flow across areas of higher and lower human disturbance—*i*.*e*., between the GB’s Cantanhez and Cufada (higher disturbance) and between sampling sites in SEN’s PNNK (lower disturbance). GB66 included 37 males and 74 females that were sampled up to the maximum distance of 65.7 km (*i*.*e*., the distance between the furthest neighbouring sites) (Table A in S4 Appendix) at 12 of the 17 sampling sites of GB165 (Figure A in S1 Appendix). Five sampling sites of GB165 *i*.*e*., Cabedu in GB_Cantanhez n = 8, Guebambol in GB_Cufada n = 3, and Boé Beli, Boé Aicum and Boé Aicum Montanha in GB_Boé, n = 21, were excluded from the GB66 dataset because the sites were located more than 71 km to the farthest sampling sites in GB’s Cantanhez and Cufada (S1 Appendix). SEN66 included 97 males and 68 females sampled at five sampling sites in PNNK (Table 1). GB_Boé was excluded from the comparative analyses to SEN because the distances between sampling sites and the sampling size were not comparable to the other sub-sets (S1 Appendix).

To compare the spatial genetic structure between the sexes for GB66 and SEN66 dataset, statistical analyses were repeated using the same settings as before for STRUCTURE v2.1 [49], and for spatial autocorrelation and assignment indexes estimated in GenAlEx 6.3. To estimate spatial autocorrelation patterns, samples were grouped in four distance classes of 16.5 km wide ([0–16.5[, [16.5–33[, [33–49.5[, [49.5–66 [km, starting point). The width of the distance classes was chosen among other possible combinations to allow for a direct comparison between SEN66 and GB66 sub-sets, using the same geographic distances and including a sufficient number of pairs of individuals in each class to perform the analyses. The distance classes of [0–16.5 [and [16.5–33 [km represent pairwise comparisons between samples collected within the limits of GB_Cantanhez, GB_Cufada and PNNK. The distance classes of [33–49.5 [and [49.5–66 [km represent pairwise comparisons across the area located between GB_Cantanhez and GB_Cufada protected areas, which has a higher degree of human disturbance, and an area of a lower degree of human disturbance in SEN. Squared paired-sample t-tests (t^2^) and the omega (ω) test criteria were used as before to compare the heterogeneity between correlograms for single and overall distance classes [56]. Furthermore, we compare the spatial genetic structure of each sex between the populations for the same distance classes. *AIc*, *mAIc*, and *vAIc* were estimated for 90 males and 61 females sampled at SEN66 with no missing data.

The sub-sets of samples distanced to a maximum linear distance of 26 km are named “SEN26”, “GB_Cantanhez26” and “GB_Cufada26” (Table 1). Analyses conducted at a spatial scale to the maximum distance of 26 km compares dispersal patterns between areas of lower human disturbance within GB (*i*.*e*., within the limits of GB_Cantanhez and GB_Cufada parks, S1 Appendix) and SEN to a maximum linear distance of 26 km. Samples collected at GB_Boé were excluded from these analyses because the distances between sampling sites (*i*.*e*., 6 and 14 km) are not comparable to the other sub-sets at 26 km. SEN26 excluded Niokolo and included 82 males and 61 females from all other sampling sites. GB_Cantanhez26 and GB_Cufada26 included all sampling sites in GB66, which were divided by locality (GB_Cantanhez26: 21 males and 42 females, and GB_Cufada26: 16 males and 32 females) (S1 Appendix). We repeated the spatial autocorrelations estimated in GenAlEx 6.3 with parameters set as before to compare the spatial structure between the sexes within each locality [55]. Samples were grouped into three distance classes [0–8.66[, [8.66–17.32[, and [17.32–25.98 [km, starting point.

## Results

### Microsatellite genotyping and sex identification

The minimum average QI across loci exceeded 0.50 for both GB and SEN datasets (averaging 0.87 across loci for GB and 0.86 across loci for SEN). Mean ADO across loci was 14.2% for GB and 15.7% for SEN dataset, and mean FA across loci was 2.49% for GB and of 4.35% for SEN dataset. The probability of identity (pID) using this set of loci was 2.20 x 10^−10^ and the probability of identity among sibs (pIDsib) was 7.02 x 10^−5^. Duplicate genotypes were removed from the datasets.

For SEN and GB_Boé, a greater proportion of males than females were identified (SEN_proportion males_ = 0.59 and SEN_proportion females_ = 0.41, GB_Boé _proportion males_ = 0.62 and GB_Boé_proportion females_ = 0.38) and the opposite pattern was found in GB’s Cantanhez and Cufada (GB_Cantanhez__proportion males_ = 0.34 and GB_Cufada__proportion males_ = 0.33) (Tables A and B in S1 Appendix).

### Genetic diversity

The thirteen microsatellites exhibited three to seven alleles per locus and *He* values varied between 0.35 and 0.77 (Table A in S5 Appendix). Three and five of 13 loci were not in HWE for GB165 and SEN65 datasets, respectively (Table A in S5 Appendix). Four pairs of loci displayed significant LD for SEN65 with none for GB165 after Bonferroni correction for multiple comparisons (P < 0.001). To eliminate the possibility that population structure was the underlying cause for the lack of compliance to HWE for the SEN65 dataset, we re-tested HWE after dividing SEN65 into two groups (SI+CL+GD+LK and NK) following results obtained using STRUCTURE (see Results). Only D12S375 and D10S611 remained in HW disequilibrium for SI+CL+GD+LK and NK, respectively (Table B in S5 Appendix), which suggest that a population genetic sub-structure was responsible for HW disequilibrium.

GB and SEN displayed similar values of genetic diversity (*e*.*g*., *AR*: 4.7 for GB165 and 5.3 for SEN65, *AR* based on minimum sample size of 126 diploid individuals, Table A in S5 Appendix). When comparing between sampling localities, GB_Cantanhez had both *H*_*o*_ and *H*_*e*_ slightly lower (GB_Cantanhez: *H*_*o*_ = 0.55, *H*_*e*_ = 0.54 and other sampling localities: 0.55 < *H*_*o*_ < 0.62 and 0.59 < *H*_*e*_ < 0.60, Table C in S5 Appendix). Genetic diversity did not vary substantially between males and females in SEN and GB, although GB males showed a tendency for lower genetic diversity and more positive *F*_*is*_ values (Table C in S5 Appendix). Males in Boé possessed the lowest *H*_*o*_ (*H*_*o*_ = 0.48, Table C in S5 Appendix) and most positive *F*_*is*_ (males GB_Boé *F*_*is*_ = 0.2 and *e*.*g*. SEN males *F*_*is*_ = - 0.04, Table C in S5 Appendix). With respect to the number of effective alleles, *H*_*o*_, *H*_*e*_, *UH*_*e*_, and *F*_*is*_, females were similar across sampling locations (Table C in S5 Appendix).

### Sex-biased gene flow at a broad scale in a human-dominated environment (in GB)

**Fig 2:**
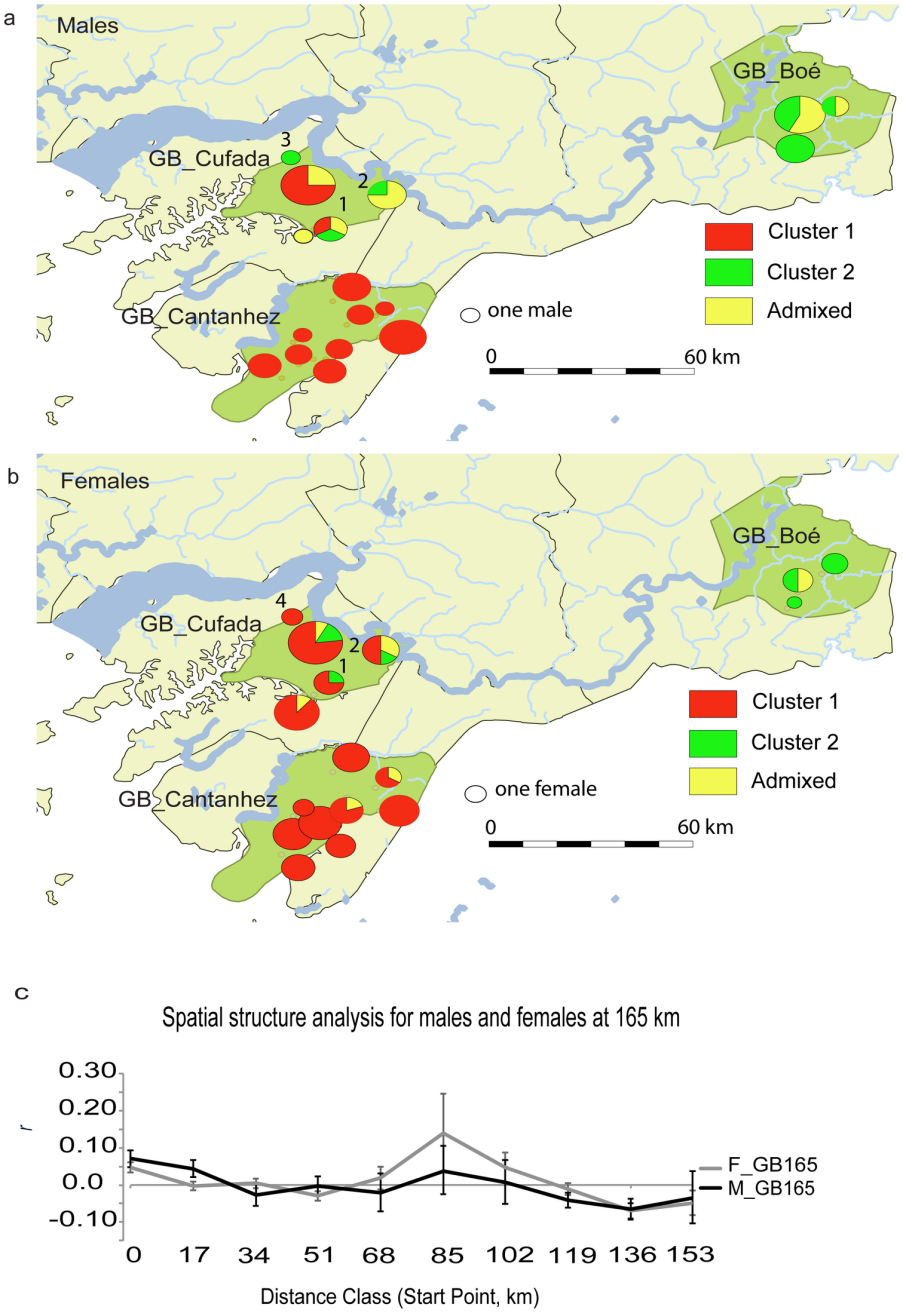
Population structure estimated in GB at 165 km. **a** and **b** shows the proportion of males (a) and females (b) at each sampling site assigned to the two STRUCTURE clusters using the GB165 sub-set. Individual-based assignment to each cluster was confirmed if q > 0.75 (cluster 1 in red and cluster 2 in green). If 0 < q < 0.75, the individual was classified as admixed between clusters (yellow). Diagram size is proportional to sample size of each site. Numbers indicate the sampling sites at GB_Cufada where individuals assigned to cluster 2 were found; 1—Sr. Soares 1, 2—Bakar Contê, 3—Guebombol, 4—Bubatchingue. **c** shows the spatial autocorrelation correlogram of the multiclass tests between females (dark grey line) and males (black line); n _pairwise comparisons_: [0–17 km[ = 1071, [17–34[ = 1183, [34–51[ = 824, [51–68[ = 899, [68–85[ = 189, [85–102[ = 22, [102–119[ = 78, [119–136[ = 656, [136–153[ = 300 and [153–170[ = 125. Males and females significantly differ in their autocorrelation pattern at the distance classes of [17–34 [(t^2^_[17–34[_ = 9.1, P = 0.002) and [119–136 [(t^2^_[119–136[_ = 4.15, P = 0.042).

When STRUCTURE was run using the GB165 sub-set, K = 2 produced the highest modal value in the ΔK distribution, with the largest Log-likelihood and with highest posterior probability (posterior probability_K2_ = 1) (Figures A and B in S6 Appendix). The plot of ranked averaged partial membership q for each individual to cluster 2 show a break between 0.71 and 0.75 and was nearly continuous between 0.75 and 1 (Figure C in S6 Appendix). Individuals were assigned to their respective genetic clusters if the averaged q > 0.75 and were considered admixed between clusters if 0 < q < 0.75. Males and females did not differ in genetic structure (Fig 2a and 2b; Figure B and Table A in S6 Appendix). For K = 2, 100% of males and 96% of females sampled at Cantanhez were assigned to cluster 1 (69 individuals, average q_cluster 1_ = 0.93, varying between 0.98 and 0.75) and two females were classified as admixed (Fig 2; Table A in S6 Appendix). In Boé, 62% of the males and 75% of the females were assigned to cluster 2 (average q_cluster 2_ = 0.86, varying between 0.95 and 0.78) or considered as admixed (Fig 2; Table A in S6 Appendix). Cufada was the only locality in which individuals were assigned to both genetic clusters; 41.2% of males and 77% of females were assigned to cluster 1 (average q_cluster 1_ = 0.91, varying between 0.97 and 0.78) and 18% of males and 12% of females were assigned to cluster 2 (average q_cluster 2_ = 0.81, varying between 0.93 and 0.75, Table A in S6 Appendix; Fig 2). Nevertheless, 41.2% of males and 12% of females were considered admixed between clusters at GB_Cufada (Table A in S6 Appendix; Fig 2).

Significant positive spatial autocorrelation was found for males and females for pairwise comparisons between neighbouring groups (*i*.*e*., at [0–17 [km or for males only at [17–34 [km, Figure D in S6 Appendix; Fig 2c). Significant negative spatial autocorrelation was found for both sexes at distances between GB_Cantanhez and GB_Cufada (at [34–51 [km and [51–68 [km) and between GB_Boé, GB_Cufada and GB_Cantanhez (males: [119–136 [and [136–153 [and females: [136–153 [and [153–170 [km) (Figure D in S6 Appendix; Fig 2c). An exception to the negative spatial autocorrelation pattern for both sexes at large distance classes comprised pairwise comparisons between GB_Boé and the sampling site Bakar Contê at GB_Cufada ([85–102 [and [102–119 [km), at which females showed significant positive autocorrelation and males show a positive but not significant autocorrelation coefficient. Nevertheless, the n _pairwise comparisons_ for the distance class [85–102 km [is particularly low for both sexes (females n = 6 and males n = 16, Figure D in S6 Appendix; Fig 2c).

The spatial autocorrelation pattern differed significantly between the sexes (Total ω for data = 21.288, P = 0.003, Fig 2c) although significance was only found in two out of the ten distance classes considered. Males are significantly more similar than females between neighbouring groups within GB_Cufada and GB_Cantanhez areas but also between few groups from GB_Cufada and GB_Cantanhez (males_[17–34 km[_: *r* = 0.04, P = 0.0002, females_[17–34 km[_: *r* = - 0.003, P = 0.3, t^2^_[17–34[_ = 9.1, P = 0.002). Females are significantly more similar than males at larger distances classes (*i*.*e*., between GB_Boé and GB_Cufada and GB_Cantanhez females _[119–136 km[_: *r* = - 0.011 and males_[119–136 km[_: *r* = - 0.041, t^2^_[119–136[_ = 4.15, P = 0.042).

When the spatial autocorrelation analyses were conducted including the samples collected at GB_Cufada and GB_Cantanhez only and excluding GB_Boé, a pattern where both sexes display significant positive spatial autocorrelation at shorter distances and significant negative spatial autocorrelation at larger distances was revealed (Figure E in S6 Appendix). *r* intercepted zero at 24 km for males, and at 14 km for females (Figure E in S6 Appendix). The spatial autocorrelation pattern did not differ between the sexes (Total ω for data = 7.834, P = 0.1, Figure F in S6 Appendix) and significant differences were found only at the [16.5–33 [km distance class, as in the previous analyses, including GB_Boé (females are more significantly dissimilar than males, females_[16.5–33[_: *r* = - 0.006, males_[16.5–33[_: *r* = 0.006, P = 0.004). However, some differences were found from the spatial autocorrelation estimated using GB165 and including GB_Boé (S6 Appendix).

GENECLASS identified six individuals as first-generation migrants using the L_h_/L_max_ statistic: one female sampled in Cantanhez with an origin in Cufada, one female sampled in Cufada with an origin in Cantanhez, two males sampled in Cufada with an origin in Boé), and two males sampled in Boé with an origin in Cufada. Of the six migrants, one male (sampled in Cufada in Sr. Soares 1 site with an origin in Boé) was identified assuming that the source population might not have been sampled. The first-generation migrant males from Boé sampled in GB_Cufada were assigned to cluster 2 in STRUCTURE analyses (q _cluster2_ = 0.93 to 0.75), which is the genetic unit mostly represented in GB_Boé (see Table C in S6 Appendix for comparison between GENECLASS results and assignment using STRUCTURE).

Males and females differed in the distribution of assignment indexes in Cufada (two-tailed U-test, P = 0.001); *mAIc* was positive for females and negative for males (*mAIc*_females_ = 0.54 and *mAIc*_males_ = -1.16), 11 out of 13 male genotypes with no missing data displayed negative *AIc* values and males showed larger *vAIc* than females (*vAIc*_females_ = 0.23 and *vAIc*_males_ = 0.37) (Table B and Figures G, H and I in S6 Appendix). *mAIc* was not significant for GB_Cantanhez (S6 Appendix).

#### Comparative analyses at a 66 km scale between GB (human-dominated environment) and SEN (lower habitat fragmentation)

When STRUCTURE was run using GB66, K = 1 was the solution with the largest Log-likelihood and with highest posterior probability (posterior probability_K1_ = 0.99) (Figure A in S7 Appendix).

For SEN66, K = 3 was the solution with the highest modal value in the ΔK distribution, with the largest Log-likelihood and with highest posterior probability (posterior probability_K3_ = 1) (Figure A in S7 Appendix). When K = 3, SEN66 is broadly clustered in GD+SI+CL+LK (cluster 1) and NK (cluster 2), and seven individuals from SI form cluster 3 (six males and one female, average q_cluster 3_ = 0.86, q varying between 0.79 and 0.93) (Figure B in S7 Appendix). We suspected that the higher genetic similarity of the seven individuals forming cluster 3 biased the analysis. After removing those samples, we re-ran STRUCTURE using parameters set as before using N = 158 genotypes (67 females and 91 males). K = 2 was found to be the most probable clustering solution (highest modal value in the ΔK distribution, the largest Log-likelihood and with posterior probability_K = 2_ = 1) (Figures C and D in S7 Appendix). The plot of ranked averaged partial membership q of each individual to clusters was discontinuous between 0.78 and 0.64 for cluster 2 and was nearly continuous between 0.68 and 1 for cluster 1 (Figure E in S7 Appendix). We assigned individuals to the respective genetic clusters if the averaged q > 0.75 and considered admixed between clusters if 0 < q < 0.75.

**Fig 3:**
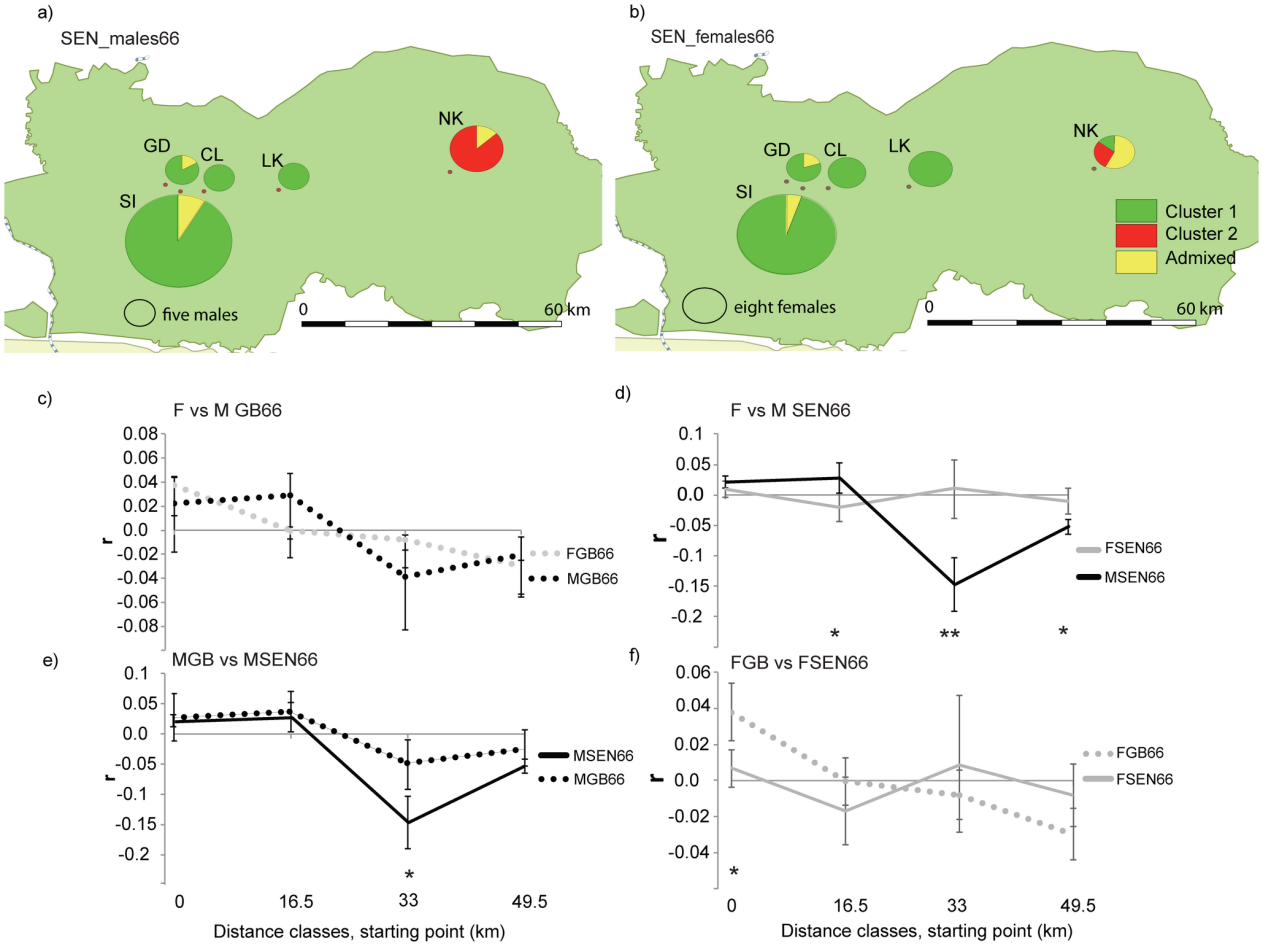
Population structure for males and females in GB and SEN at 66 km. **a** and **b** shows the proportion of males (a) and females (b) of each sampling site in Senegal (GD—Gue Damantan, SI—Simenti, CL—Camp Lion, LK—Lingue Kountou, and NK—Niokolo) assigned to each of two clusters identified by STRUCTURE (97 males and 68 females, assigned when q > 0.75 and considered admixed when 0 < q < 0.75). **c**, **d**, **e**, and **f** shows the spatial autocorrelation correlograms of the multiclass tests: c) GB females (FGB66, dashed grey line) vs. males (MGB66, dashed black line) (n _pairwise comparisons_: [0–16.5[ = 788, [16.5–33[ = 993, [33–49.5[ = 758, [49.5–66[ = 828); d) SEN females (FSEN66, bold grey line) vs. males (MSEN66 bold black line) (n _pairwise comparisons_: [0–16.5[ = 4468, [16.5–33[ = 809, [33–49.5[ = 131, [51–66[ = 1526); e) Males in GB (MGB66, dashed black line) vs. males in SEN (MSEN66, bold black line) (n _pairwise comparisons_: [0–16.5[ = 3187, [16.5–33[ = 585, [33–49.5] = 203, [49.5–66[ = 1347), and f) Females in GB (FGB66, dashed grey line) vs. females in SEN (FSEN66, bold grey line) (n _pairwise comparisons_: [0–16.5[ = 2069, [16.5–33[ = 1217, [34–49.5[ = 686, [49.5–66[ = 1007). The y-axis shows the autocorrelation coefficient *r* measuring genetic similarity (*r>0*) or dissimilarity (*r*<0) between pairs of individuals separated by four distance classes (X-axis: 0 to 49.5 km, starting point). Significant differences for individual distance classes are highlighted: *P < 0.05, **P < 0.001. The bootstrapped 95% error bars are shown. The correlagrams d and f are significantly different in the overall spatial autocorrelation pattern (d—Total ω for data = 20.21, P = 0.0001; f—Total ω for data = 11.1, P = 0.004).

Males in SEN66 showed a stronger genetic structure when compared to females (Figure D in S7 Appendix; Fig 3a and 3b). The sampling sites GD+SI+CL+LK comprise 100% of males assigned to cluster 1 (70 males, q_cluster 1 average_ = 0.96 varying between 0.99 and 0.77) and five admixed males, whereas NK harbour 100% of the males assigned to cluster 2 (q_cluster 2 average_ = 0.89 varying between 0.94 and 0.78) and two admixed males (Table A in S7 Appendix; Fig 3a and 3b). In contrast for females, NK harbour females assigned to both genetic units (cluster 1: one female, q _cluster 1_ = 0.95, cluster 2: two females, q _cluster 2 average_ = 0.83 varying between 0.87 and 0.79) and admixed individuals (Table A in S7 Appendix; Fig 3a and 3b).

For GB, no difference was detected between males and females in the global spatial autocorrelation pattern in a spatial scale of 66 km or in every single distance classes considered (Total ω for data = 4.81, P = 0.30; single classes: t^2^_[0–16.5 km[_ = 0.38, P = 0.56; t^2^_[16.5–33 km[_ = 2.29, P = 0.17; t^2^_[33–49.5[_ = 2.0, P = 0.16; t^2^_[49.5–66[_ = 0.14, P = 0.74) (Fig 3c). For both sexes, significant positive spatial autocorrelation was found for at shorter distances (*i*.*e*., [0–16.5 [km for females and [16.5–33 [km for males) and significant negative spatial autocorrelation was found at distances representing pairwise comparisons between GB_Cantanhez and GB_Cufada ([33–49.5 [and [49.6–66 [km for males and [49.5–66 [km for females) (Figure F in S7 Appendix; Fig 3c).

In contrast for SEN66, males and females differed in the spatial autocorrelation pattern (Total ω for data = 20.21, P = 0.0001; Fig 3d). In SEN, both sexes displayed significant positive spatial autocorrelation at shorter distances classes (Figure F in S7 Appendix; Fig 3d) but males were significantly more similar than females at [16.5–33 [km (t^2^_[16.5–33 km[_ = 6.63, P = 0.01; males_[16.5–33 km[_: *r* = 0.022, P = 0.0001; females _[16.5–33 km[:_ *r* = - 0.014, P = 0.008) and significantly more dissimilar at [33–66 [km (t^2^_[33–49.5 km[_ = 16.8, P = 0.0002, males_[33–49.5 km[_: *r* = - 0.11, P = 0.0001, females_[33–49.5 km[_: *r* = 0.011, P = 0.32; t^2^_[49.5–66 km[_ = 8.1, P = 0.004, males_[49.5–66 km[_: *r* = - 0.039, P = 0.0001; females_[49.5–66 km[_: *r* = - 0.008, P = 0.08) (Figure F in S7 Appendix; Fig 3d).

We compared the spatial autocorrelation of each sex between GB66 and SEN66. For males, the test for heterogeneity did not detect a difference in the overall spatial autocorrelation pattern and in three of the four single distance classes considered (Total ω for data = 5.6, P = 0.12, single classes: t^2^_[0–16.5 km[_ = 0.048, P = 0.84; t^2^_[16.5–33 km[_ = 0.085, P = 0.80; t^2^_[49.5–66[_ = 0.70, P = 0.48) (Fig 3e). However, between 33 and 49.5 km, GB66 males were significantly more similar than SEN66 males, although not reaching positive autocorrelation (t^2^_[33–49.5[_ = 6.04, P = 0.011, males GB_[33–49.5[:_ *r* = - 0.04 and males SEN_[33–49.5[:_ *r* = - 0.11). In contrast, the spatial correlogram was significantly different between females from GB66 and SEN66 (Total ω for data = 11.1, P = 0.004) (Fig 3f). When compared to SEN66 females, GB66 females were more similar at shorter distances (t^2^_[0–16.5 km[_: = 9.85, P = 0.002, *r*_females_GB_ = 0.04, *r*_females_SEN_ = 0.006).

The *mAIc* statistic did not differ significantly between males and females in SEN.

#### Comparative analyses at a 26 km scale within the limits of protected areas in GB and SEN (*i*.*e*., lower habitat fragmentation)

In SEN26, females displayed significant positive autocorrelation in distance classes representing comparisons between samples collected in neighbouring groups (females_[0–8.66[_: *r* = 0.004 P = 0.005). For GB, only females in Cantanhez26 displayed significant autocorrelation in distance classes considered; significant positive autocorrelation between neighbouring groups and significant negative autocorrelation at larger distances, which mainly includes pairwise comparisons from inside and outside the Peninsula of Cantanhez (Figure G in S7 Appendix).

## Discussion

Primate dispersal patterns may change in populations inhabiting human-dominated habitats. We assessed the directionality and extent of sex-biased gene flow using molecular data to investigate dispersal patterns in Guinea baboons (*Papio papio*) at three different geographic scales and compared two closely related populations—Guinea-Bissau (fragmented distribution, human-dominated habitat) and Senegal (continuous distribution, protected area).

The genetic population structure estimated for a large geographical scale in GB, which included three geographical distinct sub-populations populations separated by a maximum distance linear distance of 165 km (GB_Cantanhez, GB_Cufada and GB_Boé), suggested a pattern of female-mediated gene flow across large distances (Table 1). This result is in agreement with the pattern estimated using maternally transmitted mitochondrial DNA at the same geographic scale [25]: with the most frequent mtDNA haplotypes shared between GB_Cantanhez, GB_Cufada and GB_Boé, the geographic distribution of haplotypes being uncorrelated with sampling locations and significant genetic similarity being detected at larger geographic distances. Female-biased gene flow was also found for Senegal and using data of the whole distribution of the species [28, 29].

Additionally, we found evidence for contact between genetically differentiated males at GB_Cufada (Table 1). GB_Cufada was previously found to be a contact zone between genetically differentiated units [25] but in this study, we identified males sampled at GB_Cufada that could be assigned with a high probability to the genetic unit mostly represented approximately 100 km apart using STRUCTURE or that had a negative *AIc* and thus had a low probability of being assigned to the population of origin. This result contrasts with GB_Cantanhez, where all the males were assigned to the same genetic unit and 64% displayed a positive *AIc*. Furthermore, although possibly limited by a small sample size in GB_Cufada and GB_Boé (*i*.*e*., N = 51 and 21 genotypes, respectively, when 50 individuals is the minimum sample size recommended, [62]), the analyses detecting first-generation migrants identified two males sampled in Cufada with an origin in Boé and two males sampled in Boé with an origin in Cufada. The immigrant male from Boé sampled in GB_Cufada and identified assuming a more restrictive migration rate harboured a mtDNA haplotype with a high frequency in GB_Boé [25] and was assigned to the genetic unit mostly represented in GB_Boé with a high probability (*i*.*e*., q > 0.90). Taken together, these results suggest male-mediated gene flow in southern Guinea-Bissau between GB_Cufada and Boé or a genetically close population (Table 1).

The result of male-mediated gene flow in southern Guinea-Bissau should be treated cautiously. Firstly, since faecal sampling comprised unidentified individuals, we cannot be sure that the putative male immigrants dispersed during their lifetime; some males could be pre-dispersal individuals, could be the offspring of immigrant lactating females [64] or admixed individuals originated from mating between immigrant females and resident males. For instance, 41% of the males sampled in GB_Cufada were classified as having admixed ancestry, which might explain the contrast in the proportion of males identified as immigrants in different analyses (*e*.*g*. the great majority of males at GB_Cufada displayed a negative *AIc* but a smaller proportion were assigned to the differentiated cluster 2 using STRUCTURE, Fig 2). Secondly, the analyses would benefit from a larger sample of males. Although non-invasive faecal sampling of unidentified and unobserved individuals was applied in Senegal and Guinea-Bissau, a lower proportion of males were sampled in GB_Cantanhez and GB_Cufada (0.34 and 0.33, respectively) when compared to Senegal and GB_Boé (0.59 and 0.62, respectively). Since ADO between studies did not vary significantly, high ADO is unlikely to result in a failure to detect male individuals in Guinea-Bissau. A more likely explanation is the negative demographic consequences of intensive hunting practices towards males in Guinea-Bissau. Hunters have stated during interviews that they target male baboons first, due to their larger body size and consequent higher economic value [37, 65]. Male carcasses are 60% more expensive than females and other primate species at Guinea-Bissau bushmeat markets [24]. However, a lack of quantitative data from Guinea-Bissau bushmeat markets prevents us from confirming of the male-directed hunting hypothesis. Thirdly, comparison between sex-specific genetic markers (*i*.*e*., mtDNA and Y-chromosome associated markers) and autosomal microsatellites loci allows for the investigation of male-specific population structure and to explicitly test male-biased gene flow in Guinea-Bissau. However, genetic variation of the Y chromosome seems to be low in the Guinea baboon and a set of variable Y-linked chromosomal microsatellite markers is not available [33]. Nevertheless, considering that we randomly sampled double the number of females in GB_Cufada than males (34 females and 17 males, S1 Appendix) and that females and males were assigned to cluster 2 in similar proportions (12% *vs*. 18%, respectively) in that locality, our results suggest that the population of baboons at GB_Cufada comprises a proportion of genetically differentiated males (Table 1).

Our study analysed intra-specific variation in dispersal patterns using individual-based molecular tools and a comparative framework, an approach that has been applied successfully in a variety of species and landscape contexts (*e*.*g*. [13, 66–67]). However, few studies have been able to analyse direct dispersal or have attempted to compare variation in dispersal for populations under different levels of anthropogenic pressure at comparable spatial scales [68]. Molecular tools can be a solution in such cases but a combination of factors related to sampling design (inclusion of pre-dispersers in the dataset, small sample size and a different geographic distance between sites) and differences between sexes in dispersal rates can confound the detection of sex-biased dispersal [31, 32].

Here, sampling strategies were comparable between Senegal and Guinea-Bissau with respect to sample size (*e*.*g*. NGB66 = 111 and NSEN = 165), genetic markers (S5 Appendix) and geographic scale(s) but methodological constraints related to the study of wild primates may limit the generalization of our conclusions to other populations. Firstly, the inclusion of a single site in an un-fragmented population (*i*.*e*., Senegal) limits the comparison of gene flow patterns between fragmented and non-fragmented populations to a maximum of 66 km with the consequent exclusion of genotypes from the GB165 sub-set, which in turn, may have an effect in the characterization of the patterns of sex-specific gene flow. Spatial autocorrelation for different sub-sets of samples (*e*.*g*. GB165, GB_Cufada and GB_Cantanhez only, and GB66) is slightly different, although the overall pattern remained the same (Figure E in S6 Appendix). Also, the genetic structure estimated using STRUCTURE in GB165 was not detected using the GB66 sub-set, possibly because of the lower number of genetically differentiated individuals which remained in the analyses using GB66 [69]. For instance, Guebombol sampling site was excluded from the comparative analyses because was distanced more than 66 km of sampling sites in GB_Cantanhez but was formed exclusively by males assigned to the genetic unit most frequently represented in GB_Boé (Fig 2a). These results suggest that using GB66 sub-set alone can lead to incomplete inference. However, it is worth highlighting that the IUCN’s *Near threatened* Guinea baboon suffered a widespread decline in the last thirty years [35, 36] and that the current conservation status of the populations outside protected areas is unknown [70]. Therefore, the likelihood of locating a second continuously distributed population of a comparably large range in Senegal (*i*.*e*., for 165 km) is low. Secondly, the statistical power of detecting a sex-bias in dispersal is limited by the inclusion of pre-dispersing individuals in both datasets [32]. However, a clear signal of sex-biased dispersal was not erased in Senegal and it is unlikely that the Guinea-Bissau population include a significantly greater number of pre-dispersing individuals than Senegal. Therefore, we conclude that, by contrasting the differences in rate and extent of sex-mediated gene flow in Guinea-Bissau and Senegal, we are likely to be highlighting an important biological phenomenon.

For the smaller spatial scale of 66 km, for which a comparison with Senegal was performed, we could not find evidence of sex-biased dispersal in Guinea-Bissau. Both males and females at GB66 display a spatial autocorrelation pattern expected for the philopatric sex (Table 1). These results contrast with the clear pattern of female-biased dispersal found for Senegal for the same geographic scale. When comparing the spatial autocorrelation of each sex between GB66 and SEN66, we could observe that (1) males of the two populations show a similar spatial genetic pattern, although SEN66 males show a stronger genetic structure at larger distances and (2) females in Guinea-Bissau are significantly more similar than females in Senegal at closer distances.

Restricted gene flow for males and females across human-dominated areas between GB_Cufada and GB_Cantanhez do not disagree with the pattern of female-biased mediated gene flow found at a broad scale in Guinea-Bissau and the evidence of contact between genetically differentiated males in GB_Cufada. In all the spatial autocorrelation analyses performed, the pattern of limited dispersal between GB_Cufada and GB_Cantanhez is clear for both sexes. SEN66 males show a stronger genetic structure than GB66 males but we inferred hindered male gene flow between GB_Cufada and GB_Cantanhez. In Senegal, males show a genetic structure which agrees with the geographic location of sampling sites (GD+SI+CL+LK and NK, Fig 3a) whereas we found that GB_Cufada is most likely formed by mixture of genetically differentiated males, a proportion of which is genetically similar to GB_Cantanhez males (Fig 2a). This result explains the similar genetic structure pattern between males in Guinea-Bissau and Senegal but weaker genetic structure for Guinea-Bissau males at larger distances when compared to Senegal males. Taken together and considering the pattern of historical female-mediated gene flow estimated by past studies [23, 25], our study suggests a modification in dispersal patterns for the Guinea-Bissau population from a female-biased dispersal pattern at a broad geographical scale to i) male-mediated gene flow towards GB_Cufada from a differentiated population genetically similar to GB_Boé, and ii) a lack of sex bias in dispersal at a smaller scale between GB_Cufada and GB_Cantanhez.

This apparent alteration in dispersal patterns can be explained by behavioral responses to fragmentation. Anthropogenic hunting and deforestation at southern Guinea-Bissau [22, 24, 25] may have altered population density, sex ratio, and age structure and/or induced defensive behaviors, such as avoidance of specific areas and altered ranging patterns [37], changes which potentially interfere with dispersal [2, 5]. Foraging and social grouping in Guinea baboons vary according to environmental contexts [26, 71] and, most likely, to anthropogenic environmental changes, given the importance of these variables in restricting the species occurrence [70]. Other empirical studies found exceptions to the pattern defined for the target species in anthropogenic environments (*e*.*g*. *Pongo pygmaeus* [72]; *Ateles sp*. [13]), including for primate species facing the same type of threats in Guinea-Bissau (*Colobus polykomos* and *Piliocolobus temminckii* [67] and *Pan troglodytes verus* [73]). However, considering that many socio-ecological features of the Guinea baboon remain unknown (Senegal: [26, 29, 38], Guinea-Bissau: [23, 25]) we cannot exclude the possibility that the species may display population-specific plasticity in dispersal and gene flow mediated by both sexes may not be atypical. Three possible non-exclusive scenarios to explain gene flow mediated by both sexes in southern Guinea-Bissau that could be tested:

1. Firstly, if we consider that the difference in the proportion of genetically differentiated individuals co-existing in Cufada between males and females is a sampling artefact, our results could be interpreted as if entire social groups moved to the Cufada Lagoons Natural Park. One reason for the displacement of entire social groups could be the high mortality risk associated to human disturbance [74, 75], which is probable outside protected areas in Guinea-Bissau [22].
2. Secondly, while Guinea-Bissau females might be displaying the same propensity for dispersal as Senegal females, males in Guinea-Bissau may have recently started to disperse towards GB_Cufada from GB_Boé or a genetically similar population. One reason could be a lower number of males at GB_Cufada, caused by a preference to first hunt male baboons due to a higher monetary return [24, 37, 75]. The consequent reduction in number of males could lower the competition for females and food resources and thus, induce male immigration towards the vacant home ranges (“vacuum effect” [76]; *e*.*g*. *Panthera leo* [77]). Although such an effect has not been reported for primate species, sex-specific life history strategies, unequal intra-sexual competition and different sensitivity to environmental factors between the sexes have been related to a sex-bias in dispersal for other species [5]. Furthermore, past studies have associated movements of male baboons towards areas with greater accessibility to cycling females to improve their reproductive opportunities. For instance, in some populations of *Papio hamadryas*, a species in which dispersal is described to be female-biased [78], males sometimes disperse to neighbouring olive baboon groups (*Papio anubis*) due to a higher success in acquiring females [79].
3. Finally, we need to consider that the differences in dispersal patterns between Guinea-Bissau and Senegal could have been accumulated during their evolutionary history. For species that have historically expanded their range, which could be the case of the Guinea baboon [80], both theoretical considerations and simulation experiments predict a greater abundance of individuals with higher dispersal capacity at the margins of the distribution (such as in Guinea-Bissau) compared with the core (as Senegal) (*e*.*g*. [81, 82]). The effect of spatial gradients of dispersal capacity may last for a considerable amount of time after the species is settled in the new range [82]. Thus, our results may also imply an intrinsic higher dispersal capacity of the Guinea-Bissau individuals as a response to changes in the environment, a characteristic that may have been selected by evolutionary mechanisms. Data on dispersal patterns from other marginal populations are needed to test this hypothesis.

Our results do not suggest an absence of gene flow but female dispersal between GB_Cufada and GB_Cantanhez seem to have been modestly or recently restricted when compared to the same geographic scale in Senegal. One first generation female migrant with an origin in GB_Cufada sampled in GB_Cantanhez was detected, which suggest current gene flow between those localities. However, that specific female was sampled in the marginal area of Cantanhez Woodlands National Park. Significant genetic dissimilarity between females from inside and outside the Peninsula of Cantanhez (Figure G in S7 Appendix) suggest lower levels of gene flow across the land bridge connecting the Peninsula to mainland Guinea-Bissau. Although levels of inbreeding do not seem to be particularly high, GB_Cantanhez show a tendency of lower levels of genetic diversity (Table C in S5 Appendix). Overall, these results suggest a modest level of isolation of the baboons’ population from the Peninsula of Cantanhez, which may have important implications for their conservation. Considering the long generation times of primate species, the genetic consequences of a recent restriction in gene flow between GB_Cufada and GB_Cantanhez could have just started to manifest.

### Conservation recommendations

The long-term persistence of populations inhabiting human-fragmented landscapes depends on the ability of individuals to move between fragments and to reproduce [7, 83]. Particularly for species in which dispersal is female-biased, such as the Guinea baboon, fragmentation, and isolation of groups across the humanized landscape outside protected areas in GB, can greatly limit female's movements between groups [2, 3, 14] and potentially lead to a reduction of gene flow between sub-populations or even isolation. As baboons are already rarely seen outside protected areas and habitat loss and hunting practices are prone to increase, gene flow between GB_Cantanhez and GB_Cufada could become more restricted and most importantly, GB_Cantanhez could become isolated. Currently, large areas of croplands and a number of villages occupy the narrow land bridge that connects Cantanhez Peninsula to GB mainland [84]. Local villagers persecute and hunt primates and other species to avoid production losses in croplands [84] (MJ Ferreira da Silva, personal observation). Such activities have the potential to become a barrier to dispersing individuals leaving or entering the peninsula. Isolated populations have a higher risk of extinction; if mating opportunities became limited to kin and inbreeding increases, isolated populations may suffer from a reduction of reduction of reproductive fitness [7, 10, 85]. To avoid the isolation of the GB_Cantanhez population, we recommend to GB governmental agencies to strictly protect an ecological corridor located in the north of Cantanhez that allows dispersing individuals to penetrate or leave the peninsula. The Cantanhez population should be monitored in the future to detect possible negative consequences of the restriction of gene flow among protected areas. Our results support the initiative of Guinea-Bissau governmental agencies to protect ecological corridors between protected areas but emphasize that the protection of the habitat alone might not be enough to assure long-term conservation of hunted species when, at the same time, illegal hunting and extinction of groups is not prevented.

### Conclusions and future work

We have shown intraspecific variability in dispersal patterns in a primate species that is likely caused by anthropogenic fragmentation. Our results suggest some behavioral plasticity of the species to adjust to new environmental conditions. However, the severity of anthropogenic pressures could potentially overwhelm its ability to persist. We highlight that the genetic consequences of altered dispersal patterns should be evaluated more regularly and considered more thoroughly in conservation management plans.

Future work could investigate dispersal patterns for larger distances than 66 km in un-fragmented habitats. Moreover, long-distanced dispersal patterns could be investigated in other locations to assess whether the pattern of contact between genetically differentiated males is common. Given that the Guinea baboon is distributed across an increasingly smaller and fragmented area in West African countries [35] and that localized extintions are probable for the Guinea baboon populations occurring outside protected areas [23, 36], we stress the importance of studies conducted outside protected areas.

## Supporting information

S1 Appendix Sampling sites and genotypes analysed. (PDF)

S2 Appendix Description of PCRs protocols and genotyping process. (PDF)

S3 Appendix Sex-determination protocol. (PDF)

S4 Appendix Description of sub-sets of samples used to test sex-bias in dispersal. (PDF)

S5 Appendix Genetic diversity. (PDF)

S6 Appendix Sex-specific genetic structure. (PDF)

S7 Appendix Spatial autocorrelation analyses conducted at 66 and 26 km. (PDF)

## References

[1] Greenwood P. Mating systems, philopatry and dispersal in birds and mammals. Anim Behav. 1980;28:1140–62.

[2] Clutton-Brock TH, Lukas D. The evolution of social philopatry and dispersal in female mammals. Mol Ecol. 2012;21:472–92. doi: 10.1111/j.1365-294X.2011.05232.x 21883582 10.1111/j.1365-294X.2011.05232.x

[3] Lawson Handley LJ, Perrin N. Advances in our understanding of mammalian sex-biased dispersal Mol Ecol. 2007;8:1559–78. doi: 10.1111/j.1365-294X.2006.03152.x 17402974 10.1111/j.1365-294X.2006.03152.x

[4] Strier KB. Primate Behavioural Ecology. third ed New York: Pearson and Allyn and Bacon; 2007.

[5] Bowler DE, Benton TG. Causes and consequences of animal dispersal strategies: relating individual behaviour to spatial dynamics. Biol Rev. 2005;80:205–25. 15921049 10.1017/s1464793104006645

[6] Matthysen E. Density-dependent dispersal in birds and mammals. Ecography. 2005;28:403–16.

[7] Chaine A, Clobert J. Dispersal In: Candolin U, Wong B, editors. Behavioral responses to a changing world. Oxford: Oxford University Press; 2012 p. 63–79.

[8] Allendorf FW, England P, Luikart G, Ritchie PA, Ryman N. Genetic effects of harvest on wild animal populations. Trends Ecol Evol. 2008;23(6):327–33. doi: 10.1016/j.tree.2008.02.008 18439706 10.1016/j.tree.2008.02.008

[9] Radespiel U, Bruford M. Fragmentation genetics of rainforest animals: insights from recent studies. Conserv Genet. 2014;15:245–60. doi: 10.1007/s10592-013-0550-3

[10] Ferreira da Silva MJ, Bruford MW. Genetics and Primate Conservation In: Fuentes A, editor. The International Encyclopedia of Primatology. online: JohnWiley & Sons, Inc; 2017.

[11] Sugiyama Y. Socioecological factors of male chimpanzee migration at Bossou, Guinea. Primates. 1999;40(1):61–8. doi: 10.1007/BF02557702 23179532 10.1007/BF02557702

[12] Hsu M, Lin J. Troop size and structure in free-ranging Formonsan Macaques (Macaca cyclopis) at Ml. Longevity, Taiwan. Zool Stud. 2001;40(1):49–60.

[13] Di Fiore A, Link A, Schmitt CA, Spehar SN. Dispersal patterns in sympatric woolly and spider monkeys: integrating molecular and observational data Behaviour. 2009;146:437–70.

[14] Isbell LA, van Duren V. Differential Costs of Locational and Social Dispersal and their consequences for female group-living primate. Behaviour. 1996;133:1–36.

[15] Cowlishaw C, Dunbar R. Primate Conservation Biology. Chicago: The University of Chicago Press; 2000.

[16] DiBattista J. Patterns of genetic variation in anthropogenically impacted populations. Conserv Genet. 2008; 9(1):141–56.

[17] Ellsworth D, Honeycutt R, Silvy N, Bickham J, Klimstra W. Historical biogeography and contemporary patterns of mitochondrial DNA variation in white-tailed deer from the southeastern United States. Evolution. 1994;48:122–36. doi: 10.1111/j.1558-5646.1994.tb01299.x 28567797 10.1111/j.1558-5646.1994.tb01299.x

[18] Andreasen AM, Stewart KM, Longland WS, Beckmann JP, Forister M. Identification of source-sink dynamics in mountain lions of the Great Basin. Mol Ecol. 2012;21:5689–701. doi: 10.1111/j.1365-294X.2012.05740.x 22934825 10.1111/j.1365-294X.2012.05740.x

[19] Nyakaana S, Abe EL, Arctander P, Siegismund HR. DNA evidence for elephant behaviour breakdown in Queen Elizabeth National Park, Uganda. Animal Conservation. 2001;4:231–7.

[20] Jedrzejewski W, Branicki W, Veit C, Medugorac I, Pilot M, Bunevich A, et al Genetic Diversity and relatedness within packs in an intensely hunted population of wolves Canis lupus. Acta Theriol. 2005;50(1):3–22.

[21] Gobush KS, Mutayoba BM, Wasser SK. Long-Term Impacts of Poaching on Relatedness, Stress Physiology, and Reproductive Output of Adult Female African Elephants. Conserv Biol. 2008 doi: 10.1111/j.1523-1739.2008.01035.x 18759771 10.1111/j.1523-1739.2008.01035.x

[22] Casanova C, Sousa C. Plano de acção nacional para a conservação das populações de chimpanzés, cólubus vermelhos ocidentais e cólubis brancos e pretos ocidentais na República da Guiné-Bissau. Bissau: IBAP, 2007.

[23] Ferreira da Silva M, Casanova C, Godinho R. On the western fringe of baboons distribution: mitochondrial D-loop diversity of Guinea baboons (*Papio papio*, Desmarest 1820) (Primates: Cercopithecidae, Papio) in Coastal Guinea-Bissau, West Africa. Journal of Threatened Taxa. 2013;5(10):441–4450. http://dx.doi.org/10.11609/JoTT.o3216.4441-50

[24] Minhós T, Wallace E, Ferreira da Silva M, Sá R, Carmo M, Barata A, et al DNA Identificantion of primate bushmeat from urban markets in Guinea-Bissau and its implications for conservation. Biol Conserv. 2013;(167):43–9. http://dx.doi.org/10.1016/j.biocon.2013.07.018.

[25] Ferreira da Silva M, Godinho R, Casanova C, Minhós T, Sá R, Bruford MW. Assessing the impact of hunting pressure on population structure of Guinea baboons (Papio papio) in Guinea-Bissau. Conserv Genet. 2014;15:1339–55. doi: 10.1007/s10592-014-0621-0

[26] Galat-Luong A, Galat G, Hagell S. The social and ecological flexibility of Guinea baboons: implications for Guinea baboons social organization and male strategies In: Swedell L, Leigh SR, editors. Reproduction and Fitness in Baboons Behavioral, Ecological, and Life History Perspectives. New York: Springer; 2006.

[27] Renaud P, Gueye M, Hejcmanova P, Antoninova M, Samb M. Inventaire aérien et terrestre de la faune et relevé des pressions au Parc National du Niokolo Koba. Dakar, Senegal: Parc National du Niokolo Koba, 2006 Contract No.: General report.

[28] Kopp G, Ferreira da Silva M, Fischer J, Brito J, Regnaut S, Roos C, et al The Influence of Social Systems on Patterns of Mitochondrial DNA Variation in Baboons. International Journal of Primatology. 2014;351(1):210–25. doi: 10.1007/s10764-013-9725-5 24523566 10.1007/s10764-013-9725-5 PMC3915079

[29] Kopp G, Fischer J, Patzelt A, Roos C, Zinner D. Population genetic insights into the social organization of Guinea baboons (Papio papio): Evidence for female-biased dispersal. Am J Primatol. 2015;77(8):878–89. doi: 10.1002/ajp.22415 25864569 10.1002/ajp.22415 PMC4654240

[30] Bronikowski AM, Alberts SC, Altmann J, Packer C, Dee Carey K, Tatar M. The aging baboon: Comparative demography in a non-human primate. PNAS. 2002;99(14):9591–5. doi: 10.1073/pnas.142675599 12082185 10.1073/pnas.142675599 PMC123185

[31] Goudet J, Perrin N, Waser P. Tests for sex-biased dispersal using bi-parentally inherited genetic markers. Mol Ecol. 2002;11(6):1103–14. 12030985 10.1046/j.1365-294x.2002.01496.x

[32] Prugnolle F, de Meeus T. Inferring sex-biased dispersal from population genetic tools: a review. Heredity. 2007;88:161–5. 10.1038/sj.hdy.6800060 11920116

[33] Teixeira. Landscape genetics of Guinea baboons: assessign population structure, gene flow dynamics, and functional connectivity with molecular spatial tools: Universidade do Porto; 2016.

[34] Kopp GH. Gene Flow Dynamics in Baboons—The Influence of Social Systems–. Gottingen: Georg-August-Universität; 2015.

[35] Oates J, Gippoliti S, Groves C. Papio papio2008; <www.iucnredlist.org>. Downloaded on 03 January 2012.

[36] Galat G, Galat-Luong A, Keita Y. Régression de la distribuition et statut actuel du babouin Papio papio en limite d’aire de repartition au Senegal. African Primates. 1999–2000;4(1 & 2):69–70.

[37] Ferreira da Silva M. Hunting Pressure and the population genetic patterns and sex-mediated dispersal in the Guinea Baboon in Guinea-Bissau. Cardiff: Cardiff University; 2012.

[38] Patzelt A, Zinner D, Fickenscher G, Diedhiou S, Camara B, Stahl D, et al Group Composition of Guinea Baboons (Papio papio) at a Water Place Suggests a Fluid Social Organization. International Journal of Primatology. 2011;32(3):652–68. doi: 10.1007/s10764-011-9493-z 21654901 10.1007/s10764-011-9493-z PMC3083506

[39] Bayes M, Smith K, Alberts SC, Altmann J, Bruford MW. Testing the reliability of microsatellite typing from faecal DNA in the savannah baboon. Conserv Genet. 2000; 1:173–6.

[40] Roeder A, Bonhomme M, Heijmans C, Bruford M, Crouau-Roy B, Doxiadis G, et al A large panel of microsatellite markers for genetic studies in the infra-order Catarrhini. Folia Primatol. 2009;80:63–9. doi: 10.1159/000211121 19352089 10.1159/000211121

[41] Broquet T, Petit E. Quantifying genotyping errors in noninvasive population genetics. Mol Ecol. 2004;13(11):3601–8. doi: 10.1111/j.1365-294X.2004.02352.x 15488016 10.1111/j.1365-294X.2004.02352.x

[42] Waits LP, Luikart G, Taberlet P. Estimating the probability of identity among genotypes in natural populations: cautions and guidelines Mol Ecol. 2001;(10):249–56 11251803 10.1046/j.1365-294x.2001.01185.x

[43] Petit R, El Mousadik A, Pons O. Identifying populations for conservation on the basis of genetic markers. Conserv Biol. 1998;12:844–55.

[44] Goudet J. FSTAT: a program to estimate and test gene diversities and fixation indices.Version 2.9.3.2. http://wwwunilch/izea/softwares/fstathtml. 2002.

[45] Peakall R, Smouse PE. GENALEX 6: genetic analysis in Excel. Population genetic software for teaching and research. Mol Ecol Notes. 2006; 6:288–95. 10.1093/bioinformatics/bts460 PMC3463245 22820204

[46] St.George D, Witte SM, Turner TR, Weiss ML, Phillips-Conroy J, Smith EO, et al Microsatellite Variation in Two Populations of Free-Ranging Yellow Baboons (Papio hamadryas cynocephalus). International Journal of Primatology. 1996;19(2):273–85.

[47] Tung J, Charpentier MJE, Garfield DA, Altmann J, Alberts SC. Genetic evidence reveals temporal change in hybridization patterns in a wild baboon population. Mol Ecol. 2008;17. 10.1111/j.1365-294X.2008.03723.x 18363664

[48] Fontanillas P, Pettit E, Perrin N. Estimating sex-specific dispersal rates with autosomal markers in hierarchically structured populations. Evolution. 2004;58(4):886–94. 15154563 10.1111/j.0014-3820.2004.tb00420.x

[49] Pritchard JK, Stephens M, Donnelly P. Inference of Population Structure Using Multilocus Genotype Data. Genetics. 2000;155:945–59. 10835412 10.1093/genetics/155.2.945 PMC1461096

[50] Hubisz MJ, Falush D, Stephens M, Pritchard JK. Inferring weak population structure with the assistance of sample group information. Molecular Ecology Resources. 2009;9(5):1322–32. doi: 10.1111/j.1755-0998.2009.02591.x Epub 2009 Apr 1. 21564903 10.1111/j.1755-0998.2009.02591.x PMC3518025

[51] Evanno G, Regnaut S, Goudet J. Detecting the number of clusters of individuals using the software STRUCTURE: a simulation study. Mol Ecol. 2005;14 2611–20. doi: 10.1111/j.1365-294X.2005.02553.x 15969739 10.1111/j.1365-294X.2005.02553.x

[52] Earl DA, vonHoldt BM. STRUCTURE HARVESTER: a website and program for visualizing STRUCTURE output and implementing the Evanno method. Conservation Genetics Resources. 2012 doi: 10.1007/s12686-011-9548-7

[53] Beaumont M, Barratt E, Gottelli D, al. e. Genetic diversity and introgression in the Scottish wildcat. Mol Ecol. 2001;10:319–36. 11298948 10.1046/j.1365-294x.2001.01196.x

[54] Bergl RA, Vigilant L. Genetic analysis reveals population structure and recent migration within the highly fragmented range of the Cross River gorilla (Gorilla gorilla diehli) Mol Ecol. 2007;16:501–16. doi: 10.1111/j.1365-294X.2006.03159.x 17257109 10.1111/j.1365-294X.2006.03159.x

[55] Smouse PE, Peakall R. Spatial autocorrelation analysis of individual multiallele and multilocus genetic structure. Heredity. 1999;82:561–73. 10383677 10.1038/sj.hdy.6885180

[56] Smouse P, Peakall R, Gonzales E. A heterogeneity test for fine-scale genetic structure. Mol Ecol. 2008;17:3389–400. 18677808 10.1111/j.1365-294x.2008.03839.x

[57] Banks SC, Peakall R. Genetic spatial autocorrelation can readily detect sex-biased dispersal. Mol Ecol. 2012 doi: 10.1111/j.1365-294X.2012.05485.x 22335562 10.1111/j.1365-294X.2012.05485.x

[58] Beck N, Peakall R, Heinsohn R. Social constraint and an absence of sex-biased dispersal drive fine-scale genetic structure in white-winged choughs. Mol Ecol. 2008;17:4346–58. 19378407 10.1111/j.1365-294x.2008.03906.x

[59] Piry S, Alapetite A, Cornuet J., DP D, Baudouin L, Estoup A. GeneClass2: A Software for Genetic Assignment and First-Generation Migrant Detection. J Hered. 2004;95:536–9. doi: 10.1093/jhered/esh074 15475402 10.1093/jhered/esh074

[60] Paetkau D, Slade R, Burden M, Estoup A. Genetic assignment methods for the direct, real-time estimation of migration rate: a simulation-based exploration of accuracy and power. Mol Ecol. 2004;13:55–65. 14653788 10.1046/j.1365-294x.2004.02008.x

[61] Rannala B, Mountain JL. Detecting immigration by using multilocus genotypes. Proc Natl Acad Sci U S A. 1997; 94:9197–201. 9256459 10.1073/pnas.94.17.9197 PMC23111

[62] Paetkau D, Slade R, Burden M, Estoup A. Direct, real-time estimation of migration rate using assignment methods: a simulation-based exploration of accuracy and power. Mol Ecol. 2004;13:55–65. 14653788 10.1046/j.1365-294x.2004.02008.x

[63] Favre F, Balloux F, Goudet J, Perrin N. Female-biased dispersal in the monogamous mammal Crocidura russula: evidence from field data and microsatellite patterns. Proc R Soc Lond B Biol Sci. 1997; 264:127–32. 10.1098/rspb.1997.0019 PMC1688234 9061966

[64] Schubert G, Stoneking C, Arandjelovic M, Boesch C, Eckhardt N, Hohmann G, et al Male-mediated gene flow in patrilocal primates. PLoS ONE 2010;6 e21514. 10.1371/journal.pone.0021514 PMC3128582 21747938

[65] Amador R, Casanova C, Lee P. Ethnicity and perceptions of bushmeat hunting inside Lagoas de Cufada Natural Park (LCNP), Guinea-Bissau. Journal of Primatology. 2014;3(121).

[66] Pérez-Espona S, Pérez-Barbería FJ, Jiggins CD, Gordon IJ, Pemberton JM. Variable extent of sex-biased dispersal in a strongly polygynous mammal Mol Ecol. 2010;19:3101–13. doi: 10.1111/j.1365-294X.2010.04733.x 20629954 10.1111/j.1365-294X.2010.04733.x

[67] Minhós T, Nixon E, Sousa C, Vicente L, Silva MFd, Sá R, et al Genetic evidence for spatio-temporal changes in the dispersal patterns of two sympatric African Colobine monkeys. Amer J Phys Anthrop. 2013;150:464–74. doi: 10.1002/ajpa.22223 23359253 10.1002/ajpa.22223

[68] Driscoll D, Banks S, Barton P, Ikin K, Lentini P, Lindenmayer D, et al The Trajectory of Dispersal Research in Conservation Biology. Systematic Review. PLoS ONE 2014;9(4):e95053 doi: 10.1371/journal.pone.0095053 24743447 10.1371/journal.pone.0095053 PMC3990620

[69] Kalinowski ST. The computer program STRUCTURE does not reliably identify the main genetic clusters within species: simulations and implications for human population structure. Herediy. 2011;106(4):625–32. doi: 10.1038/hdy.2010.95 20683484 10.1038/hdy.2010.95 PMC3183908

[70] Vale C, Ferreira da Silva M, Campos J, Torres J, Brito J. Applying species distribution modelling to the conservation of an ecologically plastic species (Papio papio) across biogeographic regions in West Africa. Journal for Nature Conservation 2015;27:26–36. doi: 10.1016/j.jnc.2015.06.004

[71] Patzelt A, Kopp G, Nda I, Kalbitzer U, Zinner D, Fischer J. Male tolerance and male–male bonds in a multilevel primate society. Proc Natl Acad Sci U S A. 2014;111:14740–5. doi: 10.1073/pnas.1405811111 25201960 10.1073/pnas.1405811111 PMC4205614

[72] Goossens B, Chikhi L, Ancrenaz M, Lackman-Ancrenaz I, Andau P, Bruford M. Genetic Signature of Anthropogenic Population Collapse in Orang-utans PLoS Biol. 2006;4(2):e25 doi: 10.1371/journal.pbio.0040025 16417405 10.1371/journal.pbio.0040025 PMC1334199

[73] Borges F. A country-level genetic survey of the IUCN critically endangered western chimpanzee (Pan troglodytes verus) in Guinea-Bissau. Porto, Portugal: Universidade do Porto; 2017.

[74] Alberts SC, Altmann J. Immigration and Hybridization Patterns of Yellow and Anubis Baboons In and Around Amboseli, Kenya. American Journal Primatology. 2001;53:139–54. 10.1002/ajp.1 11283975

[75] Cá A. Estudos Sobre Caça e Mercado de Primatas em Tombali, Sul da Guiné-Bissau Minas Gerais: Universidade Federal de Minas Gerais; 2008.

[76] Ji W, Sarre SD, Aitken N, Hankin RKS, Clout MN. Sex-biased dispersal and a density-independent mating system in the Australian brushtail possum, as revealed by minisatellite DNA profiling. Mol Ecol. 2001;10:1527–37. 11412373 10.1046/j.1365-294x.2001.01287.x

[77] Loveridge A, Searle A, Murindagomob F, Macdonald D. The impact of sport-hunting on the population dynamics of an African lion population in a protected area Biol Conserv. 2007;134:548–58.

[78] Hapke A, Zinner D, Zischler H. Mitochondrial DNA variation in Eritrean hamadryas baboons (Papio hamadryas hamadryas): life history influences population genetic structure. Behav Ecol Sociobiol. 2001;50:483–92.

[79] Wooley-Barker T. Social organization and genetic structure in a baboon hybrid zone. New York, USA: New York University; 1999.

[80] Jolly C. Fifty years of looking at human evolution: backward, forward, and sideways. Curr Anthrop. 2009;50(2):187–99. 10.1086/597196 19817222

[81] Phillips B, Brown G, Shine R. Life-history evolution in range-shifting populations. Ecology. 2010;91(91):1617–27. 20583704 10.1890/09-0910.1

[82] Cobben M, Verboom J, Opdam P, Hoekstra R, Jochem R, Smulders M. Spatial sorting and range shifts: Consequences for evolutionary potential and genetic signature of a dispersal trait. Journal of Theoretical Biology. 2015;(373):92–9. doi: 10.1016/j.jtbi.2015.03.019 25817038 10.1016/j.jtbi.2015.03.019

[83] Clobert J, Galliard J-FL, Cote J, Meylan S, Massot M. Informed dispersal, heterogeneity in animal dispersal syndromes and the dynamics of spatially structured populations. Ecol Lett. 2009;12:197–209. doi: 10.1111/j.1461-0248.2008.01267.x 19170731 10.1111/j.1461-0248.2008.01267.x

[84] Temudo MP. The narrative of environmental degradation in Southern Guinea—Bissau: an ethnographic deconstruction. Etnográfica. 2009;13(2):237–64.

[85] Frankham R, Ballou J, Briscoe D. Introduction to Conservation Genetics. Cambridge: Cambridge University Press; 2002.

