# Correction: Disrupted dispersal and its genetic consequences: Comparing protected and threatened baboon populations (*Papio papio*) in West Africa

**DOI:** 10.1371/journal.pone.0205302

**Published:** 2018-10-01

**Authors:** Maria Joana Ferreira da Silva, Gisela H. Kopp, Catarina Casanova, Raquel Godinho, Tânia Minhós, Rui Sá, Dietmar Zinner, Michael W. Bruford

The Data Availability statement for this paper is incorrect. The correct statement is: The data underlying this study have been uploaded to the Open Science Framework and are accessible using the following link: https://osf.io/6evq2/.

The following information is missing from the Acknowledgments section: The primer sequences contained in S3 Appendix were provided to the authors by Dr. Christian Roos.
