# Supplementary material for: Disrupted dispersal and its genetic consequences: Comparing protected and threatened baboon populations (Papio papio) in West Africa
Source: PLoS One. 2018 Apr 3;13(4):e0194189. doi: 10.1371/journal.pone.0194189 (PMC5882123; doi:10.1371/journal.pone.0194189)
Supplement: S1 Appendix — (PDF) [file pone.0194189.s001.pdf]

S1 Appendix: Sampling sites and genotypes analysed

Sampling in Guinea-Bissau was carried out at: i) nine sites in GB_Cantanhez (N = 71 genotypes, average number of genotypes per sampling site = 7.89, varying between 3 and 13, average distance between sampling sites = 15.9 km, varying between 3.2 and 32.3 km), ii) five sites at GB_Cufada (N = 51 genotypes, average number of genotypes per sampling site = 10.2, varying between 3 and 21, average distance between sampling sites = 19.9 km, varying between 2.3 and 27.8 km), and iii) three sites at GB_Boé (N = 21 genotypes, average number of genotypes per sampling site = 7, varying between 5 and 11, average distance between sampling sites = 9.6 km, varying between 6.1 and 14.3 km).

In SEN, sampling was carried out at five sampling sites in an almost continuous design (N = 165 genotypes, average number of genotypes per sampling site = 33, varying between 108 and 11, the average distance between sampling sites = 18.1 km, varying between 4.1 and 65.0 km).

Supporting Information: Disrupted dispersal and its genetic consequences: comparing protected and threatened baboon populations (*Papio papio*) in West Africa

S1 Table A: Name of sampling sites within each country and number of unique multi-locus genotypes. Sampling sites in Guinea-Bissau are numbered as in Fig A

Country	Sampling Location	Name of sampling site	Number of Genotypes
**Senegal**	Niokolo Koba National Park	Camp du Lion	11
		Gue Damantan	11
		Lingue Kountou	13
		Niokolo	22
		Simenti	108
		**Total Senegal**	165
**Guinea-Bissau**	Cantanhez	1. Porto Gandamael	13
		2. Amindara	7
		3. Cabedu	8
		4. Catomboi	10
		5. Canamina	10
		6. Caiquene	3
		7. Cambeque	6
		8. Quebo-Sutuba	4
		9. Botche-Cule	10
		**Total Cantanhez**	71
	Cufada	10. Bubatchingue	21
		11. Bakar Contê	10
		12. Guebombol	3
		13. Sr. Soares 1	7
		14. Sr. Soares 2	10
		**Total Cufada**	51
	Boé	15. Boé Beli	5
		16. Boé Aicum	11
		17. Boé Aicum Montanha	5
		**Total Boé**	21
		**Total Guinea-Bissau**	143

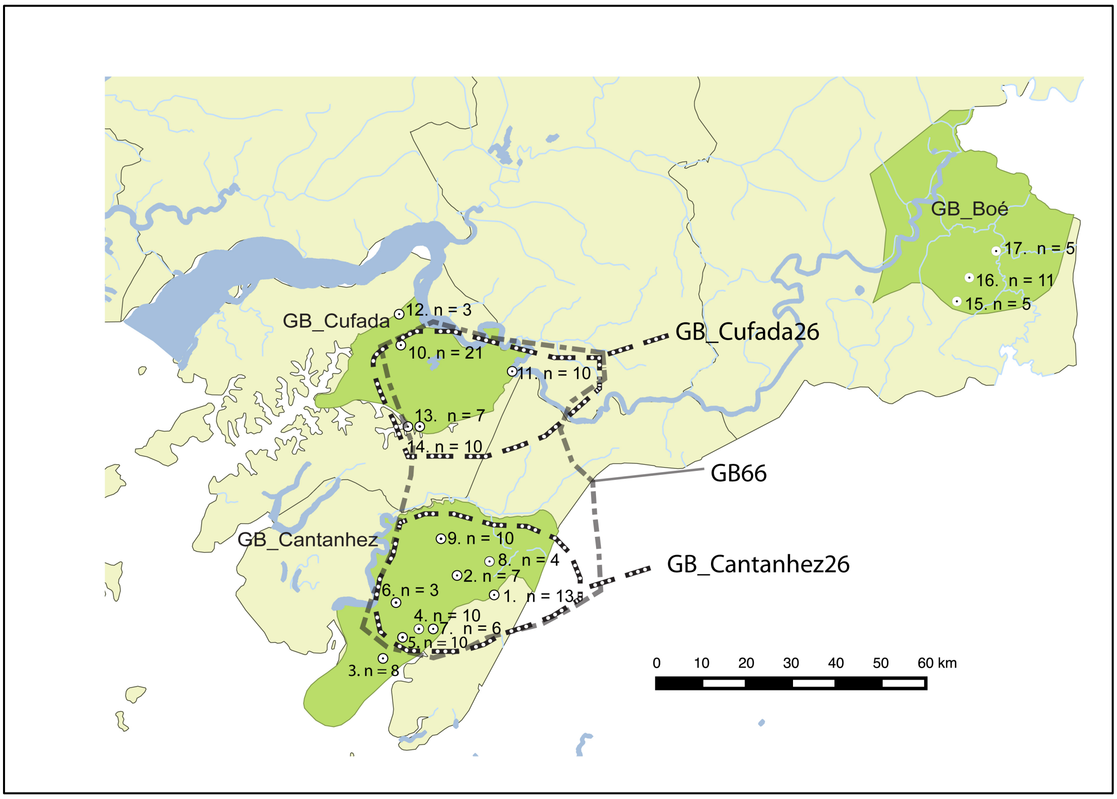

S1 Figure A. Sampling locations in GB. Sample size and grouping of samples that form sub-sets of samples in GB (GB66, GB_Cantanhez26 and GB_Cufada26). The sampling sites are numbered as in Table S1A. Produced using QGIS.

S1 Table B: **Matrix of linear distance in km between sampling sites in GB**. 1- Porto Gandamael, 2- Amindara, 3 - Cabedu, 4 Catomboi, 5 - Canamina, 6 - Caiquene, 7 - Cambeque, 8 - Quebo-Sutuba, 9 - Botche-Cule, 10 - Bubatchingue, 11- Bakar Contê, 12 - Guebambol, 13 - Sr. Soares 1, 14 - Sr. Soares 2, 15 - Boé Beli, 16 - Boé Aicum, 17 - Boé Aicum Montanha. At the top of table is indicated the name of the sub-set of samples (GB165, GB66, GB_Cantanhez26 and GB_Cufada26) and the dashed or broken vertical lines delimitate which sampling sites are included in the respective sub-set. The maximum linear distance between sampling sites is highlighted in bold. The underlined sampling sites are included in GB165 but not in GB66, GB_Cantanhez26 and GB_Cufada26.

GB165																	
GB66																	
GB_Cantanhez26									GB_Cufada26								
1	2	3	4	5	6	7	8	9	10	11	12	13	14	15	16	17	
0.0																	1
9.3	0.0																2
28.6	25.0	0.0															3
18.4	14.8	10.4	0.0														4
22.6	18.6	6.4	4.1	0.0													5
22.0	15.0	12.9	7.8	8.0	0.0												6
15.6	13.3	13.0	3.2	7.1	10.2	0.0											7
7.6	7.8	32.2	21.9	**25.9**	22.8	19.7	0.0										8
17.3	9.0	29.9	20.9	23.8	17.6	20.4	12.0	0.0									9
60.2	53.3	*70.7*	64.1	**65.9**	58.0	64.4	52.6	44.5	0.0								10
50.4	47.4	*70.6*	61.4	64.5	58.0	60.5	43.0	40.7	25.1	0.0							11
66.6	*60.1*	*77.5*	*70.9*	*72.7*	*64.9*	*71.2*	*59.1*	*51.3*	6.9	27.8	0.0						12
41.4	34.5	52.9	45.6	47.7	40.1	45.8	34.1	25.7	18.8	23.6	25.6	0.0					13
42.5	35.3	52.6	45.7	47.6	39.8	46.0	35.4	26.3	18.4	**25.9**	25.3	2.6	0.0				14
122.4	127.1	151.0	140.6	144.8	142.1	138.0	119.4	126.6	124.0	100.5	124.0	122.7	125.2	0.0			15
127.7	132.2	156.2	145.9	150.0	147.1	143.2	124.5	131.5	127.3	104.2	127.0	126.7	129.2	6.1	0.0		16
136.0	140.3	**164.5**	154.1	158.2	155.3	151.5	132.7	139.5	134.1	111.4	133.5	134.1	136.6	14.3	8.2	0.0	17

S1 Table C: Matrix of linear distances in km between sampling sites in SEN. At the top is indicated the name of the sub-set of samples (SEN66 and SEN26) and vertical lines delimitate which sampling sites are included in the respective sub-set of samples. The maximum linear distance between sampling sites is highlighted in bold.

SEN66					
SEN26					
Camp du Lion	Simenti	Gue Damantan	Lingue Kountou	Niokolo	
5.3	0.0				Simenti
8.9	3.9	0.0			Gue Damantan
17.0	22.35	**25.8**	0.0		Lingue Kountou
56.3	61.6	**65.0**	39.3	0.0	Niokolo
