# Supplementary material for: Disrupted dispersal and its genetic consequences: Comparing protected and threatened baboon populations (Papio papio) in West Africa
Source: PLoS One. 2018 Apr 3;13(4):e0194189. doi: 10.1371/journal.pone.0194189 (PMC5882123; doi:10.1371/journal.pone.0194189)
Supplement: S2 Appendix — (PDF) [file pone.0194189.s002.pdf]

S2 Appendix: Description of PCRs protocols and genotyping process

PCRs protocols

S2 Table A PCR protocols. PCR protocols followed Ferreira da Silva et al. (2014) and Koop et al. (2015). Five multiplexes PCR systems were used (M1 to M5). The annealing temperature (AT), loci identifier, GenBank code, final concentration (in µM), allelic range (base pairs) are reported.

PCRs	AT	Locus	GenBank	Final concentrations	Allelic range
M1	57 ºC	D13S765	G09003	0.2	200-212
		D12S375	G08936	0.1	164-184
		D3S1766	G08269	0.1	192-208
		D7S503	G18277	0.6	142-156
M2	55 ºC	D2S1326	G08136	0.3	192-208
		D14S306	G09055	0.2	161-181
M3	59 ºC	D8S1106	G09378	0.1	149-161
		D6S501	G08551	0.5	171-187
		D10S611	G08794	0.1	129-137
M4	57 ºC	D5S1457	G08431	0.1	125-137
		D7S2204	G08635	0.4	230-250
		D3S1768	G08287	0.1	193-212
M5*	58 ºC	D4S243	M87736	0.2	152-172
		Sex	NA	0.2	150 and/or 180

*M5 composition was slightly different for samples collected in SEN and GB. For SEN, locus D4S243 was co-amplified with locus D21S1442 (the latter was excluded from the analyses in this work). For GB, locus D4S243 was co-amplified with primers for the sex determination protocol (Sex). M2 included D1S533 (GenBank code G07788) for both SEN and GB, which was excluded from the analyses carried out in this work.

PCR cycling conditions started with a HotStarTaq DNA Polymerase activation step, during 15 min at 95ºC, followed by denaturation step at 94ºC for 30 sec,

annealing step for 40 sec at between 50ºC to 59ºC (according to each multiplex) and extension at 72ºC for 60 sec, repeated 40 cycles. The PCR ended with a final extension of 30 min at 72ºC.

Genotyping process

Genotyping followed a modified multi-tubes approach and four amplifications per locus and sample were conducted (producing 95% confidence in consensus genotypes, Ferreira da Silva et al. 2014). Only samples with a quality index (QI) (Miquel et al. 2006) above 0.50 were considered (i.e. half of the PCRs yielded the same peaks as in the consensus). A subset of GB and SEN samples were genotyped using Kopp's et al. (2015) and Ferreira da Silva's et al. (2014) amplification protocols to score alleles consistently between datasets.

Test for repeated individuals was performed using Excel microsatellite toolkit (Park, 2001) and GIMLET v1.3.3 (Valière, 2002), allowing for one mismatch or two for samples being distinguished by only one locus with a homozygote genotype.

References

Miquel C, Bellemain E, Poillot C, Bessiére J, Durand A, Taberlet P. 2006. Quality indexes to assess the reliability of genotypes in studies using noninvasive sampling and multiple-tube approach. Molecular Ecology Notes 6(4): 985–988

Broquet T, Petit E. 2004. Quantifying genotyping errors in noninvasive population genetics. Molecular Ecology 13:3601–3608.

Park, S. D. E. 2001. *Trypanotolerance in West African Cattle and the Population Genetic Effects of Selection* University of Dublin.

Valière, N. and Berthier, P. and Mouchiroud, D. and Pontier, D. 2002. Gemini: software for testing the effects of genotyping errors and multitubes approach for individual identification. Molecular Ecology Notes 2: 83-86.

Waits, L. P. and Luikart, G. and Taberlet, P. 2001. Estimating the probability of identity among genotypes in natural populations: cautions and guidelines Molecular Ecology (10): 249–256.
