# Supplementary material for: Disrupted dispersal and its genetic consequences: Comparing protected and threatened baboon populations (Papio papio) in West Africa
Source: PLoS One. 2018 Apr 3;13(4):e0194189. doi: 10.1371/journal.pone.0194189 (PMC5882123; doi:10.1371/journal.pone.0194189)
Supplement: S3 Appendix — (PDF) [file pone.0194189.s003.pdf]

S3 Appendix: Sex-determination protocol

Primer details

The molecular protocol to determine sex uses two primers designed on the Dead Box gene (F: GGACGRACTCTAGATCGGTA, R: GTNCAGATCTARGAGGAAGC). Primers amplify two fragments in the male (150 base pairs and 180 base pairs) and only one fragment in the female (180 base pairs).

PCR conditions

The sex-determination marker was amplified in a 10uL volume using the QIAGEN Multiplex PCR Kit® and 2uL of DNA extract, 1x QIAGEN Multiplex PCR Master Mix ®, 0.2uM of primer mixture and 0.75uM BSA. PCR cycling conditions started with a HotStarTaq DNA Polymerase activation step, during 15 min at 95ºC, followed by denaturation step at 94ºC for 30 sec, annealing step for 40 sec at between 58ºC and extension at 72ºC for 60 sec, repeated 40 cycles. The PCR ended with a final extension of 30 min at 72ºC. The forward primer was end-labeled with a PET fluorescent dye (Applied Biosystems) and PCR products were run on an ABI3730XL capillary analyser using a 16 GeneScanTM -500 LIZ ® size-standard. The result was confirmed if observed at least three out of four repeats.
