# Supplementary material for: Disrupted dispersal and its genetic consequences: Comparing protected and threatened baboon populations (Papio papio) in West Africa
Source: PLoS One. 2018 Apr 3;13(4):e0194189. doi: 10.1371/journal.pone.0194189 (PMC5882123; doi:10.1371/journal.pone.0194189)
Supplement: S4 Appendix — (PDF) [file pone.0194189.s004.pdf]

Supporting Information: Disrupted dispersal and its genetic consequences: comparing protected and threatened baboon populations (*Papio papio*) in West Africa

S4 Appendix: Description of sub-sets of samples used to test sex-bias in dispersal

To select sampling sites in GB to match inter-site distances in SEN, we estimated the pairwise Euclidean linear geographic distances between samples for GB and SEN separately using GenAlEx 6.3 and plotted the distribution of ranked distances (Fig. S4A).

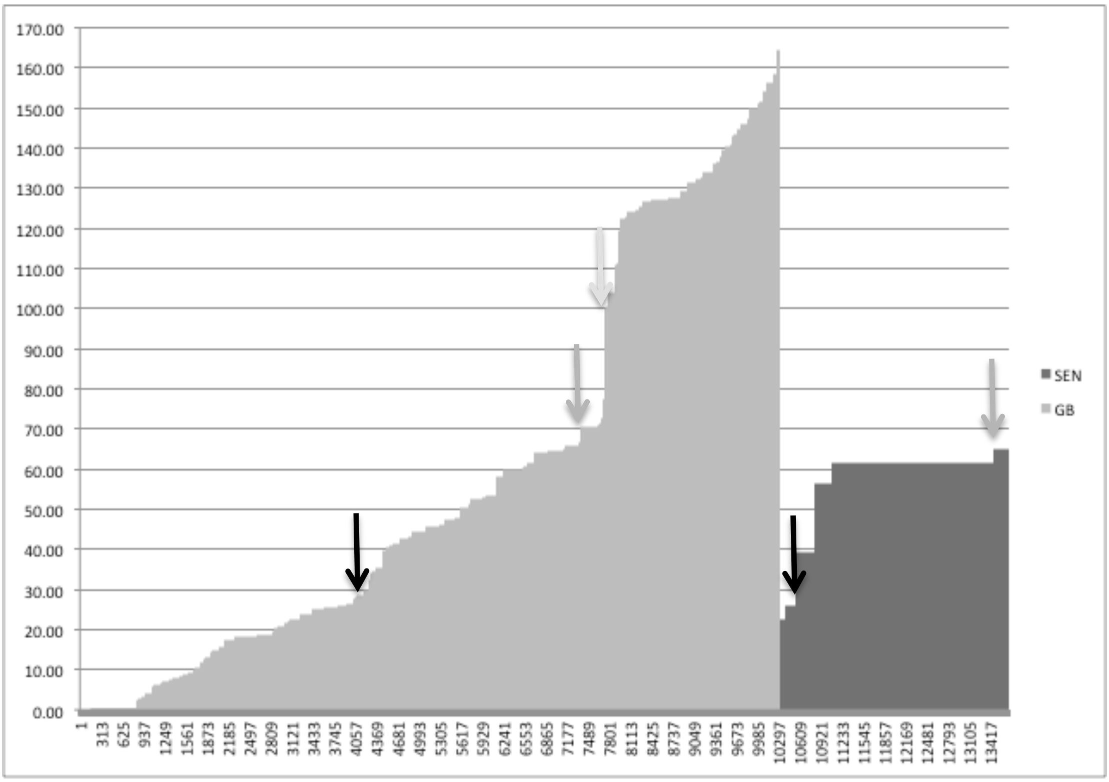

S4 Figure A: **Distribution of ranked pairwise Euclidean distances between samples collected in GB (light grey) and SEN (dark grey).** On the Y-axis is shown the Euclidean distance in km and on the X-axis is showed pairwise sample comparisons. Arrows show the gaps found in the nearly continuous distribution of pairwise distances between samples in GB: at 25.9 km (black arrow), between 65.8 km and 70.7 km (grey arrow) and between 77.6 km and 100.1 km (light grey arrow).

We could observe that the distribution of pairwise linear Euclidean distances within the geographic scale of SEN (*i.e.* < 65 km) in GB is nearly continuous but gaps can be observed between 1) 65.8 km and 70.7 km, which corresponds to the pairwise comparisons

between Cabedu in GB_Cantanhez (n= 8 genotypes) and Guebambol in GB_Cufada (n = 3 genotypes), and 2) between 77.6 km and 100.1 km, which correspond to the pairwise comparisons including the sampling sites at GB_Boé - Boé Beli (n = 5 genotypes) Boé Aicum, n = 11 genotypes, and Boé Aicum Montanha (n = 5 genotypes). Furthermore, excluding Cabedu in GB_Cantanhez and Guebambol in GB_Cufada, the maximum linear distance between sampling sites for both GB's protected areas was of 25.9 km, which had a direct correspondence in the SEN dataset after excluding Niokolo (n = 22 genotypes). The gaps at 26 km and 66 km were comparable between GB and SEN. We set the thresholds of 66 and 26 km to select sampling sites in GB and performed the comparative analyses at those distances classes.

S4 Table A: Number of males and females in the sub-set of samples. The sex was determined using a sex-based determination protocol (see S3 Appendix). In the table is indicated the name of sampling site in each population. In Guinea-Bissau, groups highlighted in bold were included in the GB165 sub-set but excluded from the GB66, GB_Cantanhez26 and GB_Cufada26. SEN66 - genotypes collected in Senegal at a maximum distance of 66 km. SEN26: genotypes collected in Senegal at a maximum distance of 26 km. GB165: genotypes collected in Guinea-Bissau at a maximum distance of 165 km. GB66: genotypes collected in Guinea-Bissau at a maximum distance of 66 km. GB_Cantanhez26 and GB_Cufada26: genotypes collected in Guinea-Bissau in Cantanhez Woodlands National Park and Cufada Lagoons Natural Park, respectively, at a maximum distance of 26 km.

**Country**	Name of site	**SEN66**			**SEN26**		
**Senegal**		M	F	Total	M	F	Total
**Niokolo Koba National Park**	Camp du Lion	5	6	11	5	6	11
	Gue Damantan	6	5	11	6	5	11
	Lingue Kountou	5	8	13	5	8	13
	Niokolo	15	7	22	-	-	-
	Simenti	66	42	108	66	42	108
	Total	97	68	165	82	61	143

		**GB165**			**GB66**			**GB_Cantanhez26**			**GB_Cufada26**		
**Guinea-Bissau**		M	F	Total	M	F	Total	M	F	Total	M	F	Total
**Cantanhez**	Porto Gandamael	6	7	13	6	7	13	6	7	13	-	-	-

	Amindara	2	5	7	2	5	7	2	5	7	-	-	-
	**Cabedu**	3	5	8	-	-	-	-	-	-	-	-	-
	Catomboi	3	7	10	3	7	10	3	7	10	-	-	-
	Canamina	2	8	10	2	8	10	2	8	10	-	-	-
	Caiquene	1	2	3	1	2	3	1	2	3	-	-	-
	Cambeque	2	4	6	2	4	6	2	4	6	-	-	-
	Quebo-Sutuba	1	3	4	1	3	4	1	3	4	-	-	-
	Botche-Cule	4	6	10	4	6	10	4	6	10	-	-	-
	Bubatchingue	8	13	21	8	13	21	-	-	-	8	13	21
	Bakar Contê	4	6	10	4	6	10	-	-	-	4	6	10
**Cufada**	**Guebombol**	1	2	3	-	-	-	-	-	-	-	-	-
	Sr. Soares 1	3	4	7	3	4	7	-	-	-	3	4	7
	Sr. Soares 2	1	9	10	1	9	10	-	-	-	1	9	10
	**Boé Beli**	4	1	5	-	-	-	-	-	-	-	-	-
**Boé**	**Boé Aicum**	7	4	11	-	-	-	-	-	-	-	-	-
	**Boé Aicum Montanha**	2	3	5	-	-	-	-	-	-	-	-	-
	Total	54	89	143	37	74	111	21	42	63	16	32	48

Supporting Information: Disrupted dispersal and its genetic consequences: comparing protected and threatened baboon populations (*Papio papio*) in West Africa
