# Supplementary material for: Disrupted dispersal and its genetic consequences: Comparing protected and threatened baboon populations (Papio papio) in West Africa
Source: PLoS One. 2018 Apr 3;13(4):e0194189. doi: 10.1371/journal.pone.0194189 (PMC5882123; doi:10.1371/journal.pone.0194189)
Supplement: S5 Appendix — (PDF) [file pone.0194189.s005.pdf]

S5 Appendix: Genetic diversity

S5 Table A: Genetic diversity per locus for the GB165 and SEN66 datasets. AR (Allelic Richness), H_o and H_e (observed and expected heterozygosity) are indicated per locus and across loci (Overall). HWE - tests for Hardy Weinberg Equilibrium deviation. NS - not significant, * P < 0.05, ** P < 0.01, *** P < 0.001. 1 GB165 includes all samples collected in Guinea-Bissau at 165 km scale. SEN66 includes all samples collected in Senegal at 66 km scale.

**Loci**	***AR***		H_o		H_E		**HWE**	
	GB165	SEN66	GB165	SEN66	GB165	SEN66	GB165	SEN66
D12S375	6	5	0.57	0.76	0.60	0.77	*	**
D7S503	5	7	0.46	0.79	0.52	0.76	NS	NS
D3S1766	5	3	0.61	0.36	0.66	0.36	NS	NS
D13S765	4	5	0.63	0.5	0.59	0.49	NS	NS
D10S611	3	4	0.30	0.65	0.35	0.53	NS	*
D6S501	5	5	0.68	0.72	0.66	0.67	NS	*
D8S1106	4	5	0.60	0.54	0.60	0.55	NS	NS
D3S1768	5	5	0.50	0.60	0.50	0.54	NS	*
D7S2204	5	6	0.64	0.74	0.60	0.74	NS	NS
D5S1457	4	4	0.51	0.50	0.53	0.43	NS	NS
D4S243	5	7	0.72	0.82	0.70	0.71	NS	***
D2S1326	4	7	0.48	0.60	0.55	0.55	*	NS
D14S306	6	6	0.69	0.54	0.78	0.58	*	NS
Overall	4.7±0.2	5.31±0.4	0.57±0.03	0.62±0.04	0.59±0.03	0.59±0.04		

S5 Table B: *Ho* and *He* (observed and expected heterozygosity) and tests for Hardy Weinberg deviation (HW) for each of the clusters detected by STRUCTURE (Delta K criterion) in SEN66. NS - not significant, * P < 0.05, ** P < 0.01. SI - Simenti, CL - Camp du Lion, GD - Gue Damantan, LK - Lingue Kountou, NK - Niokolo.

	SI+CL+GD+LK			NK		
Loci	H_o	H_e	**HW**	H_o	H_e	**HWE**
D12S375	0.76	0.77	**	0.77	0.72	NS
D7S503	0.78	0.77	NS	0.82	0.70	NS
D3S1766	0.38	0.38	NS	0.23	0.27	NS
D13S765	0.52	0.50	NS	0.41	0.42	NS
D10S611	0.61	0.52	NS	0.91	0.59	*
D6S501	0.73	0.67	NS	0.59	0.61	NS
D8S1106	0.53	0.53	NS	0.62	0.55	NS
D3S1768	0.56	0.51	NS	0.86	0.67	NS
D7S2204	0.77	0.73	NS	0.50	0.58	NS
D5S1457	0.44	0.42	NS	0.73	0.52	NS
D4S243	0.80	0.67	NS	0.91	0.75	NS
D2S1326	0.58	0.51	NS	0.65	0.59	NS
D14S306	0.53	0.57	NS	0.59	0.58	NS

S5 Table C: Genetic Diversity per sampling location. Table shows the mean over 13 microsatellite loci ± Standard error of N (genotypes), N_a (number of alleles), N_e (number of effective alleles), observed (H_o), expected (H_e) and unbiased expected heterozygosity (UH_e), coefficient of inbreeding (F_{is}) estimated per geographically distinct localities (SEN, GB_Cantanhez, GB_Cufada and GB_Boé) and separately for males and females.

Locations	*N*	N_a	N_e	H_o	H_e	UH_e	F_{is}
SEN	163.9±0.35	5.31±0.35	2.71±0.26	0.62±0.04	0.59±0.04	0.59±0.04	-0.055±0.02
GB_Cantanhez	70.1±1.1	4.15±0.22	2.39±0.24	0.55±0.04	0.54±0.04	0.55±0.04	-0.003±0.028
GB_Cufada	53.0±0.47	4.46±0.29	2.59±0.19	0.60±0.04	0.59±0.03	0.59±0.03	-0.025±0.018
GB_Boé	19.9±0.96	4.31±0.31	2.65±0.18	0.55±0.043	0.60±0.023	0.62±0.024	0.098±0.052
Males							
SEN	96.46±0.24	5.23±0.30	2.71±0.20	0.62±0.04	0.59±0.03	0.60±0.03	-0.04±0.03
GB_Cantanhez	23.3±0.31	3.69±0.21	2.20±0.19	0.51±0.04	0.51±0.04	0.52±0.04	0.00±0.04
GB_Cufada	16.54±0.18	4.15±0.25	2.79±0.16	0.59±0.03	0.62±0.02	0.64±0.02	0.06±0.03
GB_Boé	12.50±0.5	4.08±0.26	2.53±0.16	0.48±0.05	0.59±0.02	0.62±0.02	0.20±0.07
Females							
SEN	67.46±0.14	4.77±0.40	2.69±0.30	0.63±0.05	0.58±0.04	0.59±0.04	-0.08±0.02
GB_Cantanhez	44.92±0.74	4.10±0.24	2.45±0.24	0.57±0.04	0.56±0.03	0.56±0.03	-0.03±0.03
GB_Cufada	33.54±0.27	4.08±0.24	2.44±0.18	0.62±0.04	0.56±0.03	0.57±0.04	-0.12±0.04
GB_Boé	7.0±0.45	3.39±0.29	2.64±0.28	0.69±0.06	0.57±0.04	0.62±0.04	-0.21±0.08

Supporting Information: Disrupted dispersal and its genetic consequences: comparing protected and threatened baboon populations (*Papio papio*) in West Africa
