# Supplementary material for: Disrupted dispersal and its genetic consequences: Comparing protected and threatened baboon populations (Papio papio) in West Africa
Source: PLoS One. 2018 Apr 3;13(4):e0194189. doi: 10.1371/journal.pone.0194189 (PMC5882123; doi:10.1371/journal.pone.0194189)
Supplement: S6 Appendix — (PDF) [file pone.0194189.s006.pdf]

S6 Appendix: Sex-specific genetic structure

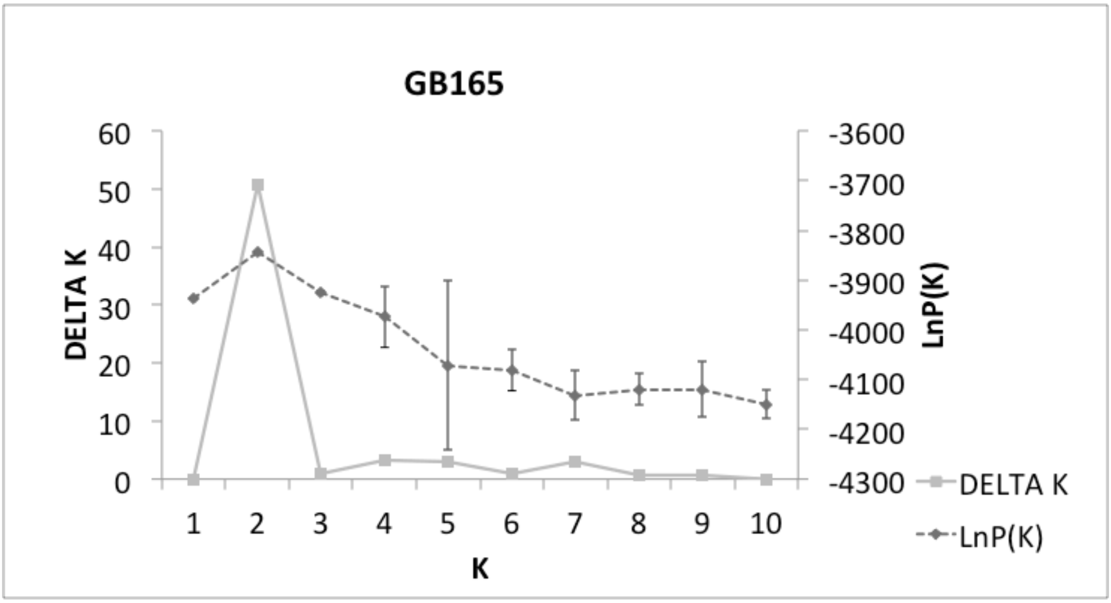

S6 Figure A Graphs show DELTA K and Mean Ln P(K) values as a function of the number of putative populations (K) for GB165. K = 2 was the solution with the highest modal value in the ΔK distribution, with the largest Log likelihood, and with highest posterior probability (of 1)

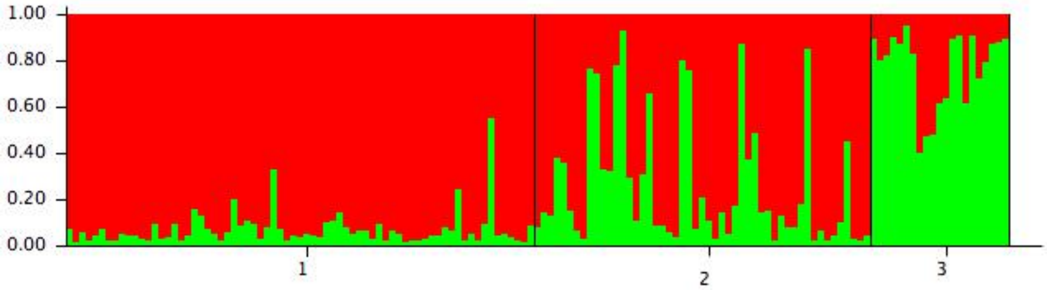

a)

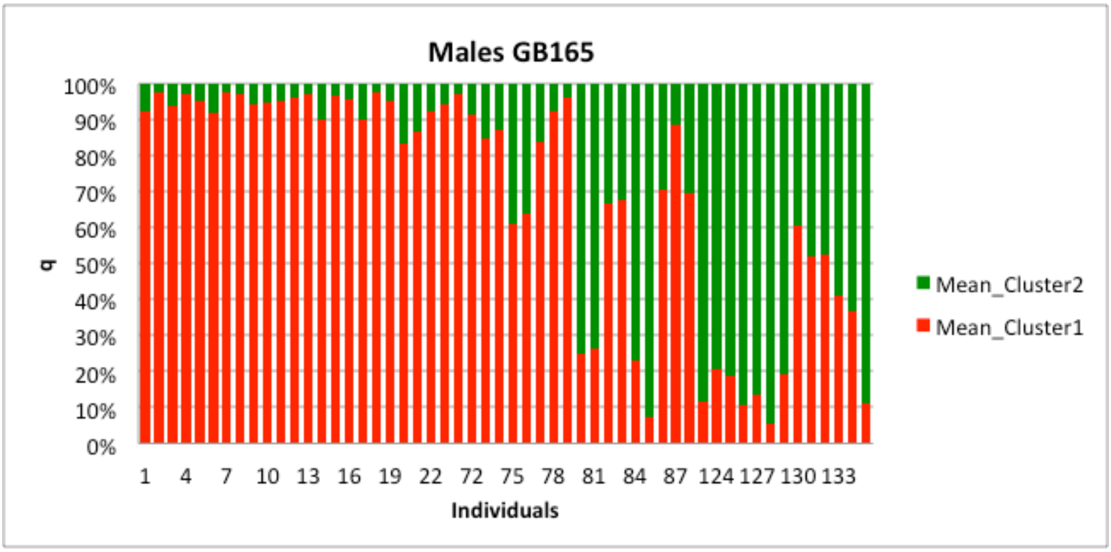

b)

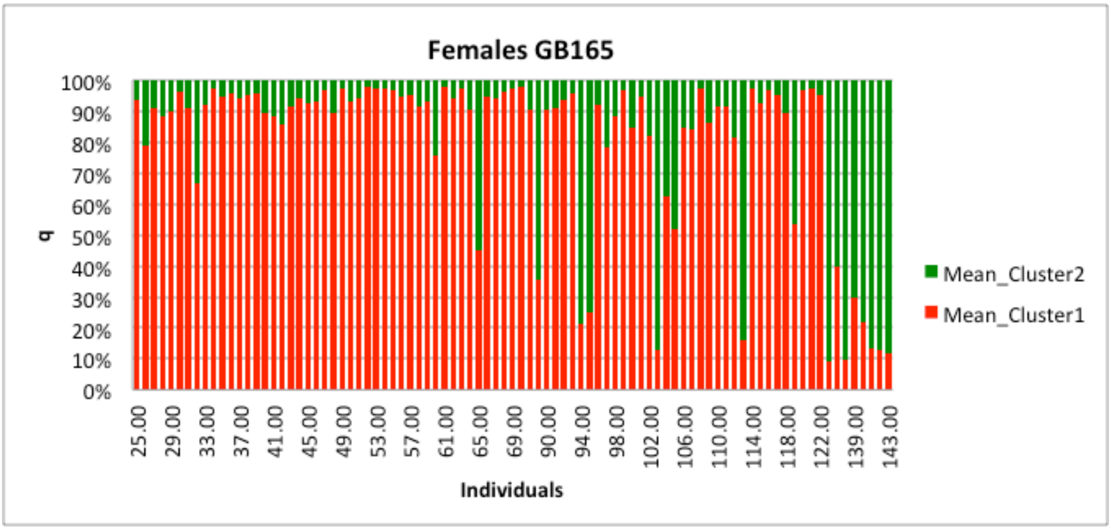

c)

Figure S6B STRUCTURE output for the GB165 subset (54 males and 89 females) when K = 2. In the graphs a single vertical bar represents an individual baboon. Y-axis indicates q membership of individuals to each of the clusters. a) K = 2 was considered the most probable clustering solution. 1 - GB_Cantanhez, 2 - GB_Cufada and 3 -

GB_Boé. b) and c) The individual probability of assignment (q) was averaged over the five independent runs carried out and the output is shown for males only (b) and for females only (c).

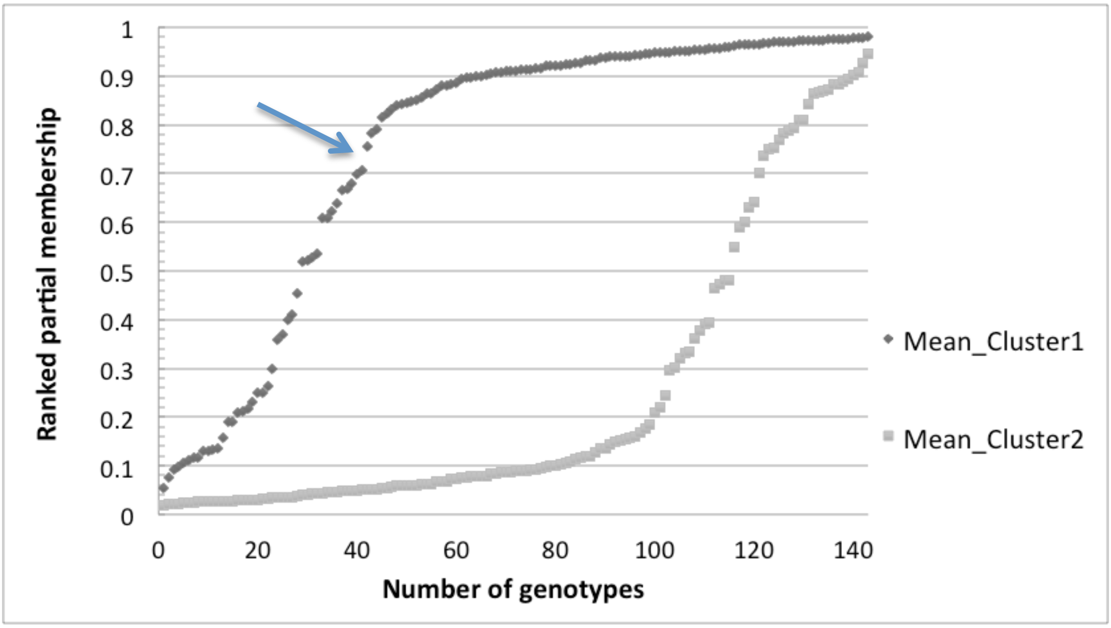

S6 Figure C Plot of ranked partial membership q of each individual to cluster 1 and 2 averaged over the five independent runs carried out. The values are almost continuous but a break was observed when q = 0.75 (indicated by the blue arrow).

S6 Table A. Assignment of GB165 individuals when K = 2 per sampling region. Individuals were assigned to each cluster when the respective individual probability of assignment q averaged across the five independent runs was > 0.75 and classified as admixed when 0 < q < 0.75. In the table is indicated the number of individuals per sampling region (N), the average, the maximum (Max), and the minimum (Min) of the individual probability of assignment q, total number of males (N M) and females (N F) and proportion of Males (M %) and females (F %) assigned to each cluster.

Sampling Region	N	Average	Max	Min	N M	N F	M (%)	F (%)
GB_Boé	21				13	8		
Cluster1	0	0	0	0				
Cluster 2	14	0.86	0.95	0.78	8	6	61.5	75.0
Admixed	7				5	2	38.5	25.0
qcluster1		0.45	0.61	0.30				
qcluster2		0.55	0.7	0.39				
GB_Cantanhez	71				24	47		
Cluster1	69	0.93	0.98	0.75	24	45	100	95.74
Cluster2	0							
Admixed	2				0	2	0	4.26
qcluster1		0.56	0.67	0.45				
qcluster2		0.44	0.55	0.33				
GB_Cufada	51				17	34		
Cluster1	33	0.91	0.97	0.78	7	26	41.18	76.47
Cluster2	7	0.81	0.93	0.75	3	4	17.65	11.76
Admixed	11				7	4	41.18	11.76
qcluster1		0.57	0.71	0.26				
qcluster2		0.43	0.74	0.29				

S6 Figure D Spatial autocorrelation for females and males using samples distanced at a maximum of 165 km in GB. a - females (N = 89 genotypes, n $_{\text{pairwise comparisons}}$ [0-17[= 812, [17-34[= 936, [34-51[= 672, [51-68[= 705, [68-85[= 143, [85-102[= 6, [102-119[= 42, [119-136[= 332, [136-153[= 182, and [153-170[= 86) and b - males (N = 54 genotypes, n $_{\text{pairwise comparisons}}$ [0-17[= 259, [17-34[= 247, [34-51[= 152, [51-68[= 194, [68-85[= 46, [85-102[= 16, [102-119[= 36, [119-136[= 324, [136-153[= 118 and [153-170[= 39). The y-axis shows the autocorrelation coefficient *r* measuring genetic similarity (*r* > *0*) or dissimilarity (*r* < 0) between pairs of individuals separated by ten distance classes (X-axis: 0 to 170 km, starting point). Significant differences are indicated in the legends of each figure.

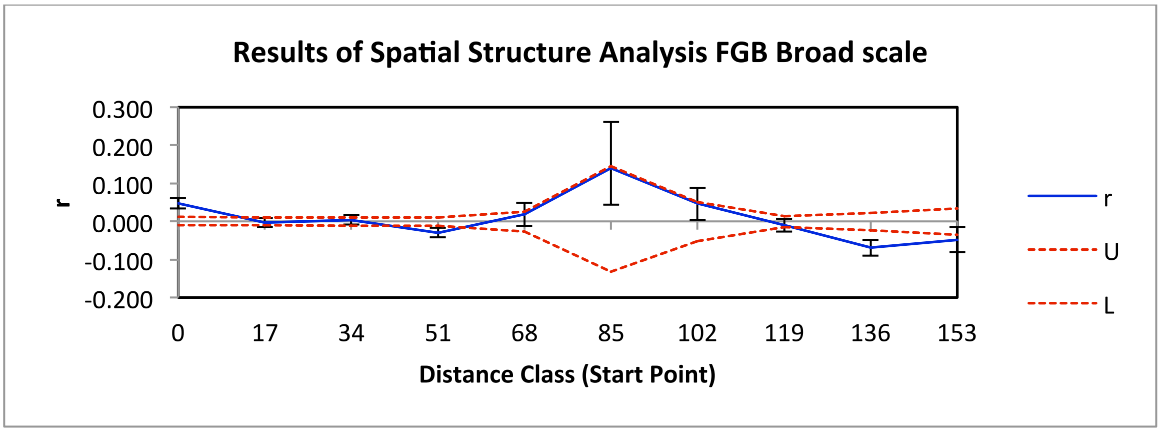

a) **Females:** Significant positive autocorrelation was found at [0-17[(P = 0.0001), [85-102[(P = 0.028), [102-119[(P = 0.038). Significant negative autocorrelation was found at [51-68[(P = 0.0001), [136-153[(P = 0.0001) and [153-170[(P = 0.004)

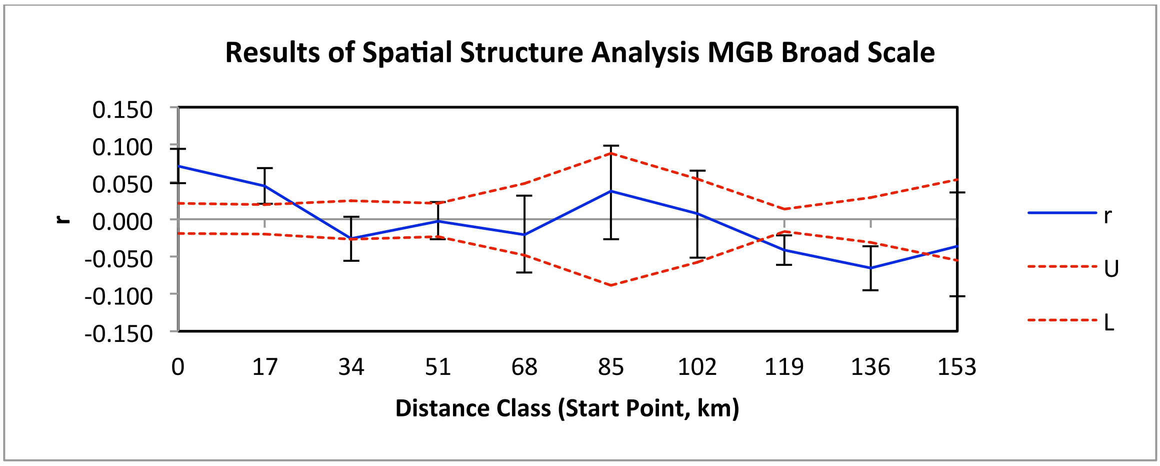

b) **Males:** Significant positive autocorrelation was found at [0-17[(P = 0.001) and [17-34[(P = 0.002). Significant negative autocorrelation was found at [34-51[(P = 0.02), [119-136[(P = 0.0002) and [136-153[(P = 0.0002)

S6 Figure E Spatial autocorrelation for females and males using GB165, divided by sampling regions (GB_Cufada and GB_Cantanhez). a - females (N = 81 genotypes, n $_{\text{pairwise comparisons}}$ [0-16.5[= 784, [16.5-33[= 936, [33-49.5[= 630, [49.5-66[= 733, [66-85] =157 and b - males (N = 41 genotypes, n $_{\text{pairwise comparisons}}$ [0-16.5[= 181, [16.5-33[= 247, [33-49.5[= 128, [49.5-66[= 212, [66-85] = 52. The y-axis shows the autocorrelation coefficient *r* measuring genetic similarity (*r > 0*) or dissimilarity (*r* < 0) between pairs of individuals separated by five distance classes (X-axis: 0 to 82.5 km, starting point). Significant differences are indicated in the legends of each figure.

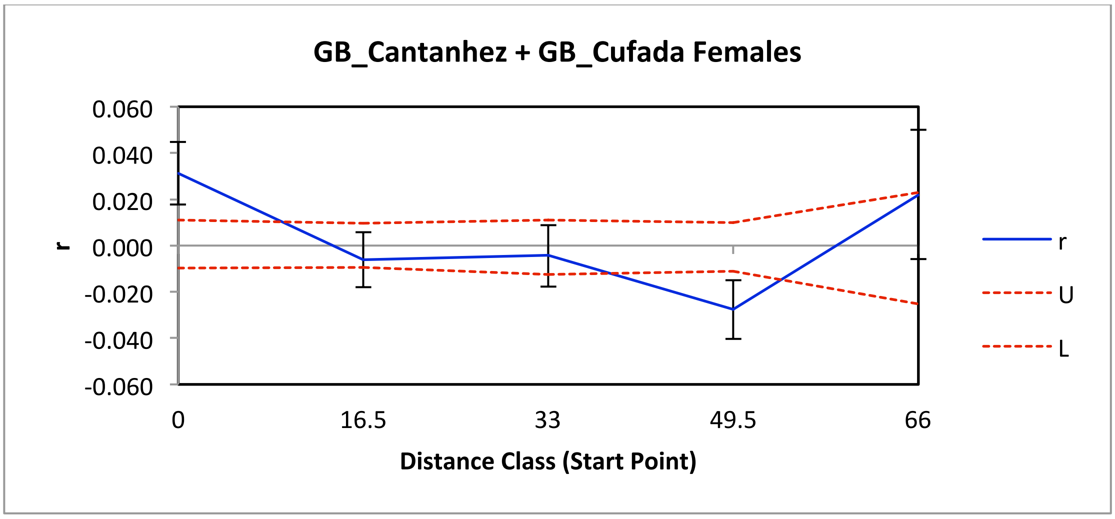

a) **Females** Significant positive autocorrelation was found at [0-16.5[(P = 0.0001) and [66-85[(P = 0.033). Significant negative autocorrelation was found at [49.5-66[(P = 0.0001).

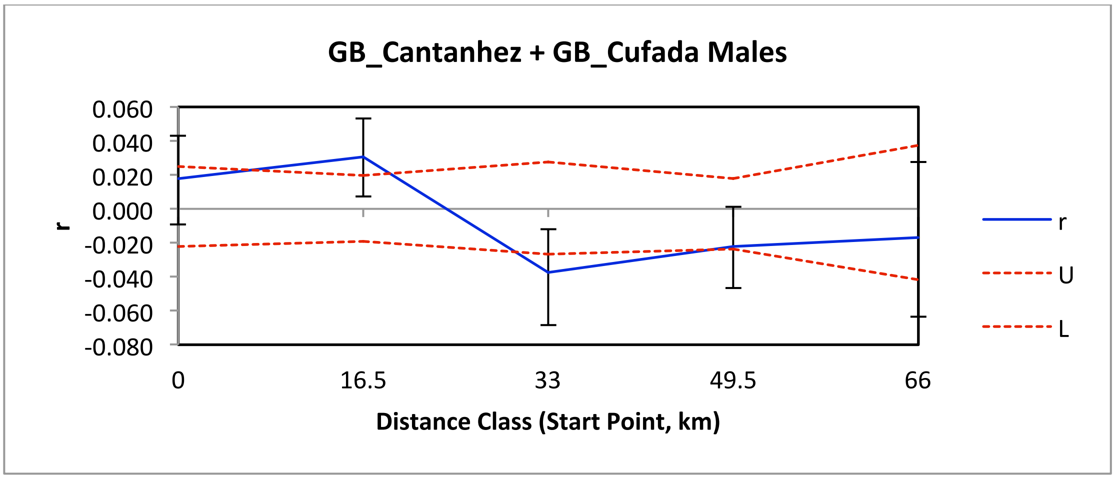

b) **Males** Significant positive autocorrelation was found at [16.5-33[(P = 0.003). Significant negative autocorrelation was found at [33-49.5[(P = 0.004) and [49.5-66[(P = 0.029)

S6 Figure F Spatial autocorrelation correlogram of the multiclass tests using GB165 divided by sampling regions (GB_Cufada and GB_Cantanhez). Females (N= = 81 genotypes) are represented by a red line and males (N = 41 genotypes) are represented by a blue line). n $_{\text{pairwise comparisons}}$ [0-16.5 km[= 965, [16.5-33[= 1183, [33-49.5[= 758, [49.5-66[= 945, [66-85[= 209. The y-axis shows the autocorrelation coefficient *r* measuring genetic similarity (*r > 0*) or dissimilarity (*r* < 0) between pairs of individuals separated by five distance classes (X-axis: 0 to 66 km, starting point). Significant differences are indicated.

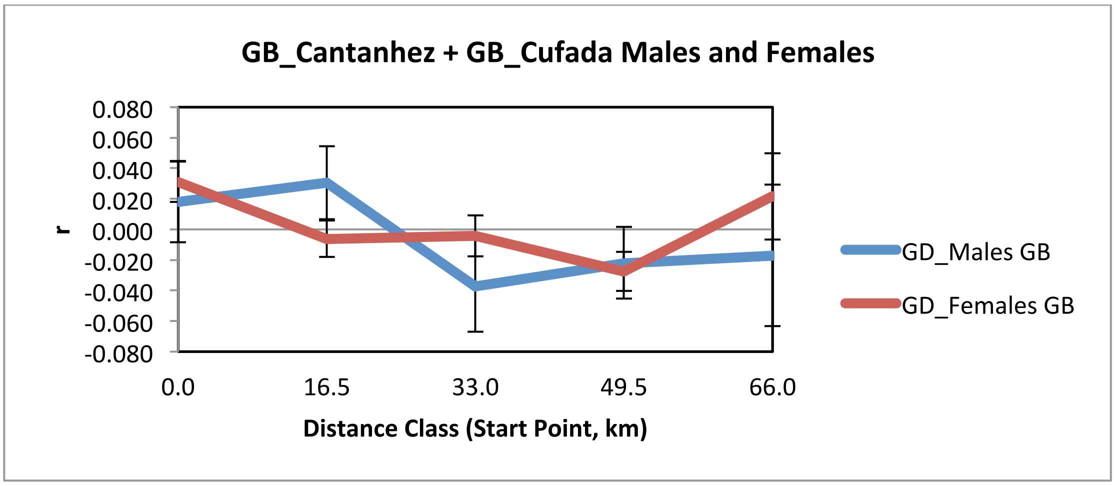

The spatial autocorrelation pattern was not different between the sexes (Total ω for data = 7.834, P = 0.1) and significant differences was only found at [16.5-33[km distance class (P = 0.004), in which females are genetically dissimilar but males are genetically similar.

Differences found between the spatial autocorrelation for females and males using GB165 and divided by sampling regions (GB_Cufada and GB_Cantanhez), and the spatial autocorrelation estimated for females and males using GB165 and including GB_Boé

i) Significant genetic similarity is only achieved in the second distance class for GB_Cufada and GB_Cantanhez males (males$_{[16.5\text{-}33\text{ km}[}$: *r* = 0.03, P = 0.002)

ii) GB_Cufada and GB_Cantanhez females reach genetic similarity at the distance class of [66-85[(females$_{[66\text{-}85\text{ km}[}$: *r* = 0.022, P = 0.033), although the number of

pairwise comparisons is particularly low for the distance class ($n_{pairwise\ comparisons}$: [0-16.5[= 181, [16.5-33[= 247, [33-49.5[= 128, [49.5-66[= 212, [66-85] = 52).

For both males and females, significant negative spatial autocorrelation was found at distances between the two protected areas ($males_{[33\text{-}49.5\ km[}$: r = - 0.04, P = 0.004 , $males_{[49.5\text{-}66\ km[}$: r = - 0.02, P = 0.03, $females_{[49.5\text{-}66\ km[}$: r = - 0.03, P = 0.001). r intercepts zero at 24 km for males at 14 km for females (Fig. S6E, S6 Appendix).

S6 Table B Test for differences in *mAIc* and *vAIc* estimated in GenAIEx using samples without missing data per sampling locations. Na - number of males (M) and females (F) without missing data used. *mAIc* - mean corrected assignment index
and *vAIc* - variance of assignment. Significant differences in *mAIc* between the sexes was found in Cufada only (two-tailed U test: two tail probability for Z, P = 0.001, Lower tailed probability for Z, P = 0.0006, Upper tailed probability for Z, non significant).

Locations	Na	*mAIc*	*vAIc*
SEN66	F (61)	0.28	0.17
	M (90)	-0.20	0.20
GB_Cufada	F (28)	**0.538**	0.23
	M (13)	**-1.16**	0.37
GB_Cantanhez	F (26)	-0.218	0.30
	M (14)	0.404	0.40

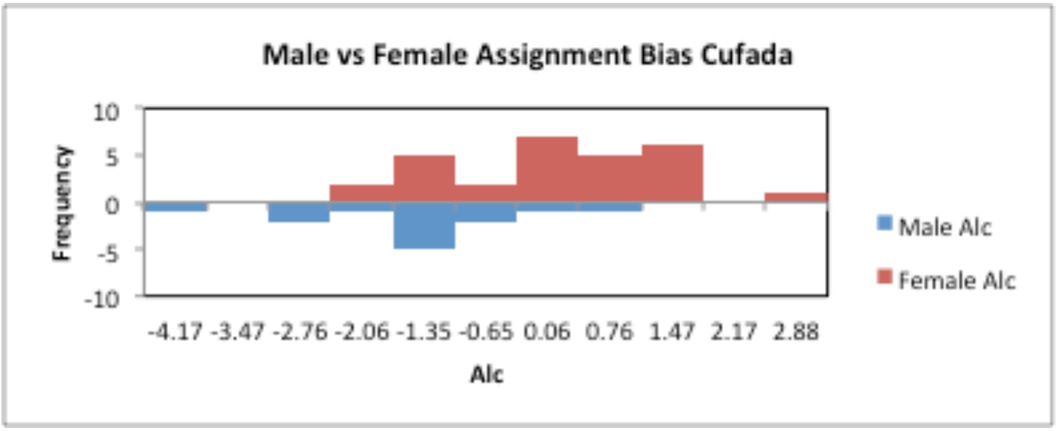

S6 Figure G Frequency Distributions of sex bias at GB_Cufada. Graph depicts the frequency of *AIc* values for samples identified as males and females at GB_Cufada. The dispersing individuals are expected to display a lower negative *AIc*.

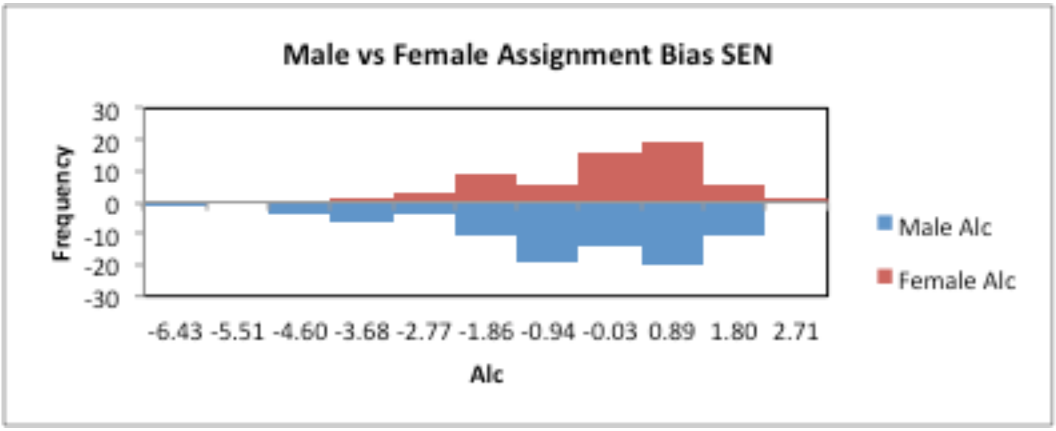

S6 Figure H Frequency Distributions of sex bias at SEN. Graph depicts the frequency of *AIc* values for samples identified as males and females at SEN. The dispersing individuals are expected to display a lower negative *AIc*.

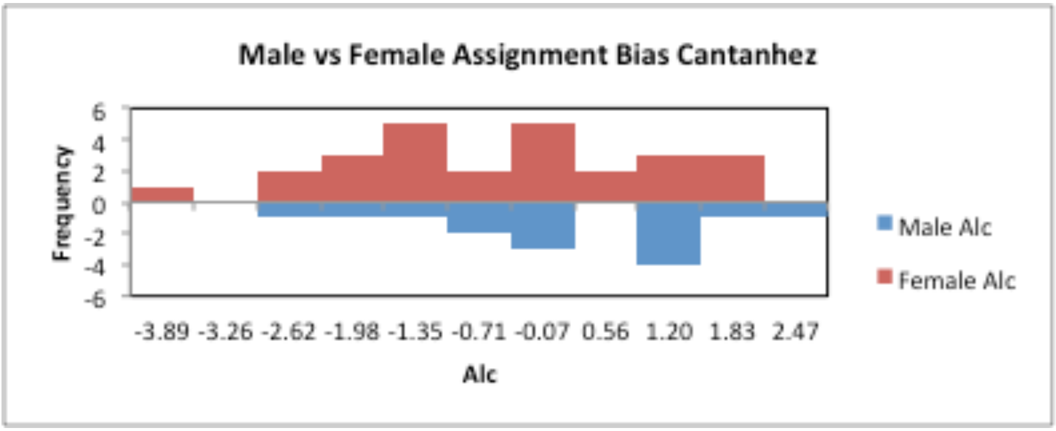

S6 Figure I Frequency Distributions of Sex Bias at GB_Cantanhez. Graph depicts the frequency of *AIc* values (x-axis) for samples identified as males and females at GB_Cantanhez. The dispersing individuals are expected to display a lower negative *AIc*.

S6 Table C Table showing the sex of samples identified by GENECLASS as first generation migrants using the Lh/Lmax and the the Lh statistic (P < 0.01). The sample identified using both statistics is highlighted in bold. The assignment of the samples by STRUCTURE analyses is indicated.

ID samples	Using the Lh/Lmax statistic	Using the Lh statistic (probabilities)	Classified by STRUCTURE analysis as	sex
CAQUES1089	p < 0.01	NS	Admixed	F
CUBAK1234	p < 0.01	NS	**Cluster 2**	**M**
**CU1SOA1307**	**p < 0.01**	**p < 0.01**	**Cluster 2**	**M**
CUBUB1217	p < 0.01	NS	Cluster 1	F
BOAIC26	p < 0.01	NS	Admixed	M
BOAIC27	p < 0.01	NS	Admixed	M
