# Supplementary material for: Disrupted dispersal and its genetic consequences: Comparing protected and threatened baboon populations (Papio papio) in West Africa
Source: PLoS One. 2018 Apr 3;13(4):e0194189. doi: 10.1371/journal.pone.0194189 (PMC5882123; doi:10.1371/journal.pone.0194189)
Supplement: S7 Appendix — (PDF) [file pone.0194189.s007.pdf]

S7 Appendix Spatial autocorrelation analyses conducted at 66 and 26 km

S7 Figure A Results from STRUCTURE analyses conducted at 66 km. a) shows the most probable clustering solution for GB66 and b) shows the most probable clustering solution for SEN66.

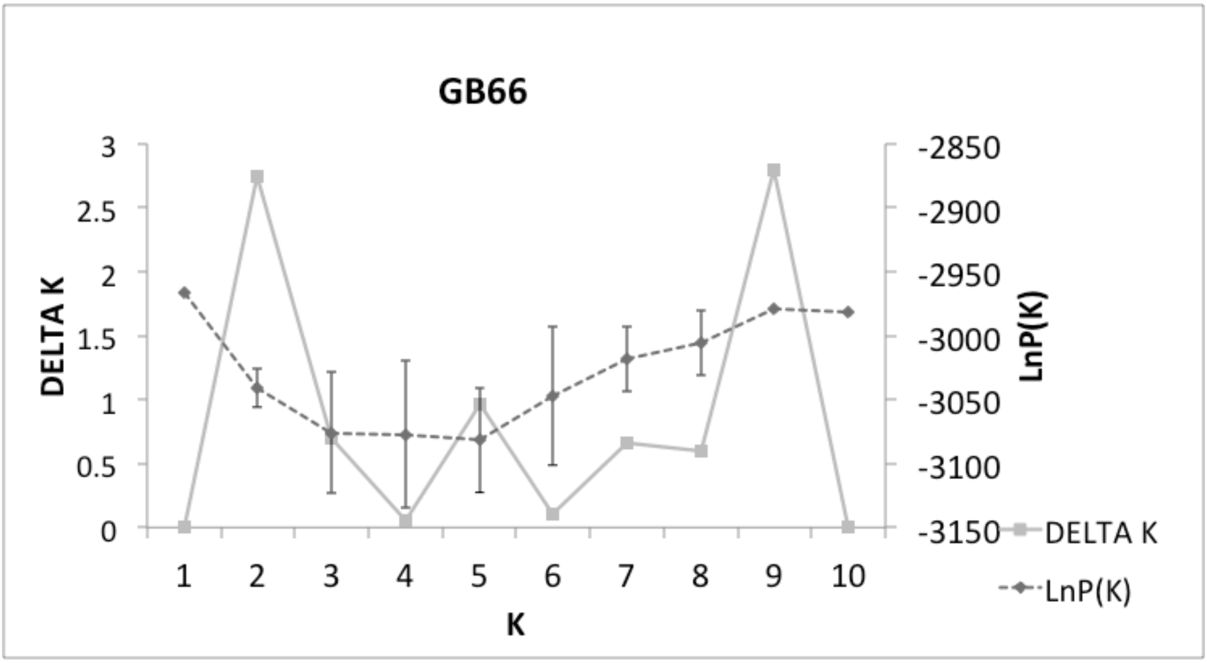

a) Graph showing K = 1 was the clustering solution with the largest Log-likelihood (LnP(K)) using GB66 dataset.

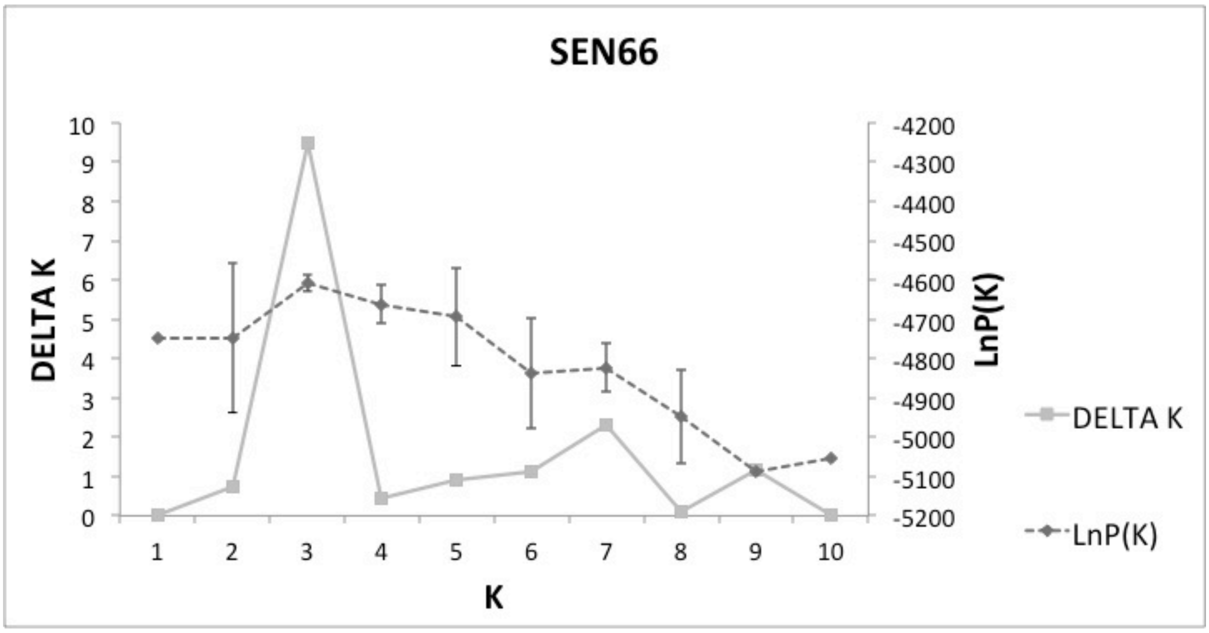

b) Graph showing K = 3 was the clustering solution with the highest modal value in the DELTA distribution and with the largest Log-likelihood (LnP(K)) using SEN66 dataset.

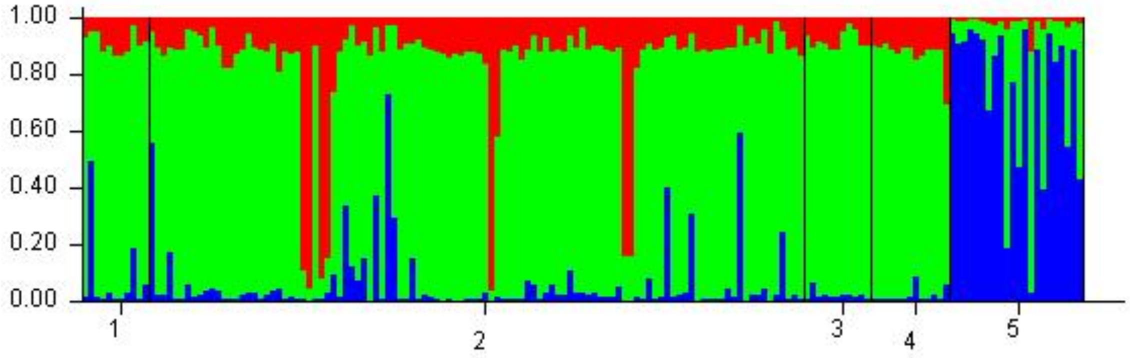

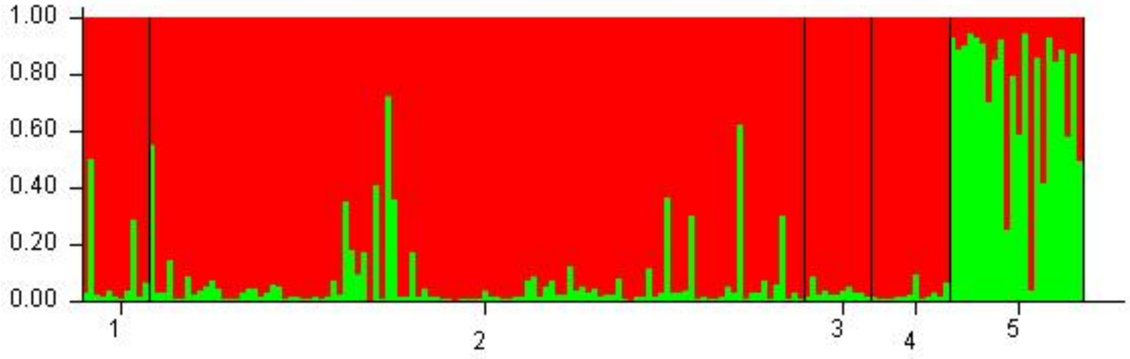

S7 Figure B STRUCTURE outputs when K = 3 (top) and K = 2 (bottom) for SEN66 (97 males and 68 females). A single vertical bar represents an individual baboon. Y-axis indicates q membership of individuals to each of the clusters. 1 - GD, 2 - SI, 3 - CL, 4 - LK and 5 - NK. For SEN66, K = 3 was the solution with the highest modal value in the ΔK distribution, with the largest Log-likelihood and with highest posterior probability (posterior probability$_{K3}$ = 1). Cluster 3 is formed by seven individuals from SI.

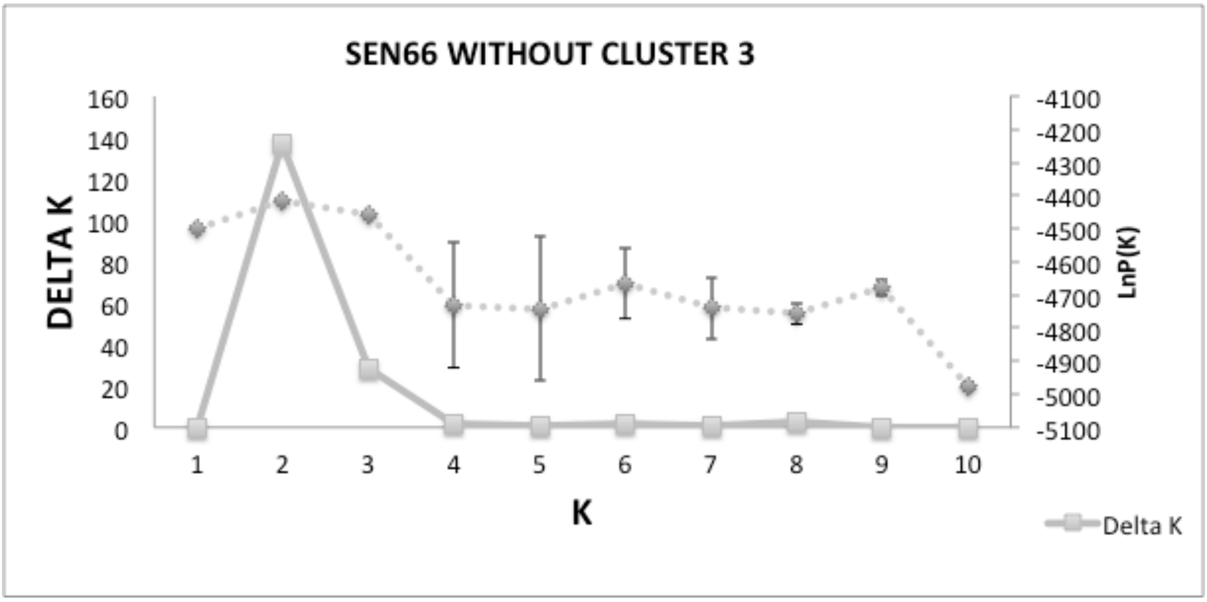

S7 Figure C Graph showing K = 2 was the clustering solution with the highest modal value in the DELTA distribution and with the largest Log-likelihood (LnP(K)) using SEN66 after removing seven individuals that formed cluster 3.

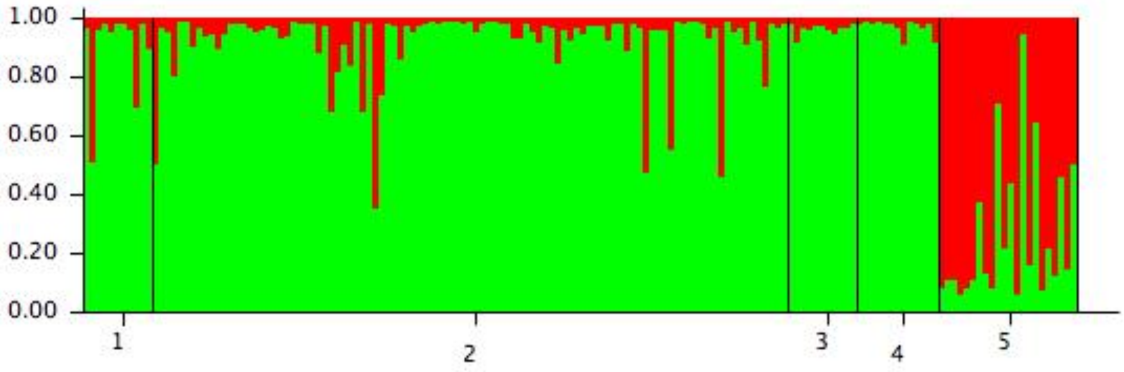

a)

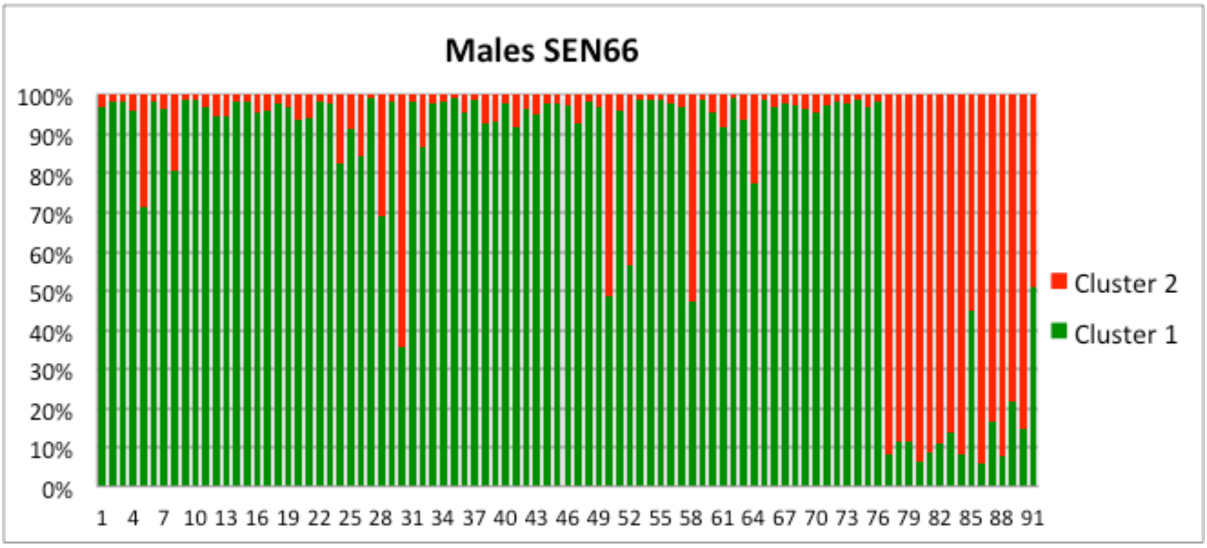

b)

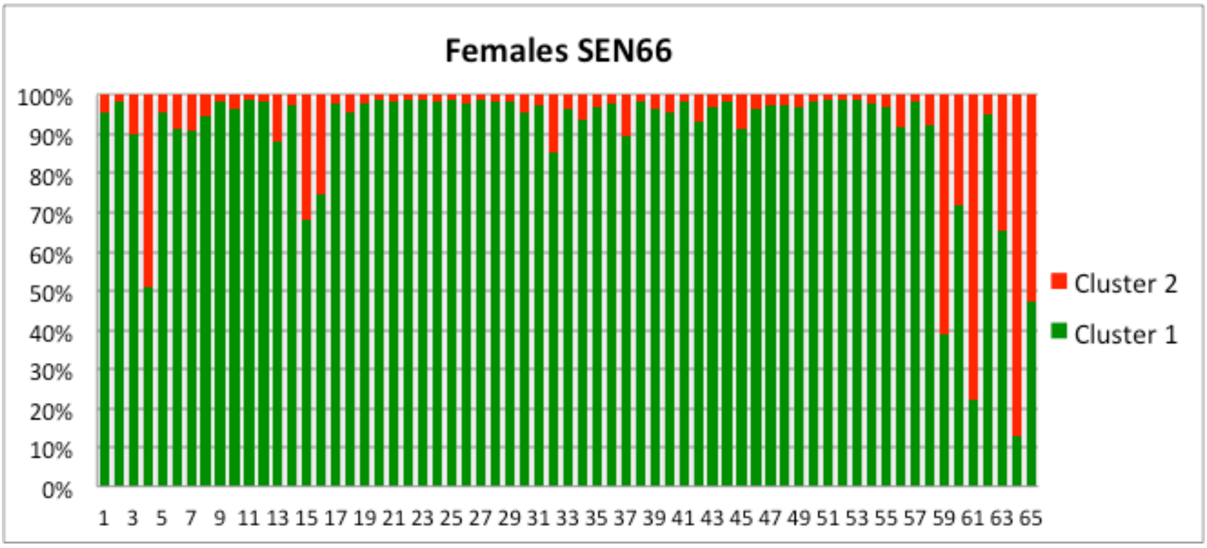

c)

S7 Figure D Graphs depicts STRUCTURE outputs when K = 2 for SEN66 (91 males and 67 females) after removing seven individuals sampled in SI which formed an artificial cluster in previous STRUCTURE runs. A single vertical bar represents an individual baboon. Y-axis indicates q membership of individuals to each of the clusters. K = 2 was the solution with the highest modal value in the ΔK distribution, with the largest Log-likelihood and with highest posterior probability (posterior probability$_{K2}$ = 1). a) Males and females together, 1 - GD, 2 - SI, 3 - CL, 4 - LK, and 5 - NK; b) and c) The individual probability of assignment (q) was averaged over the five independent runs carried out and the output is shown for males only (b) and for females only (c).

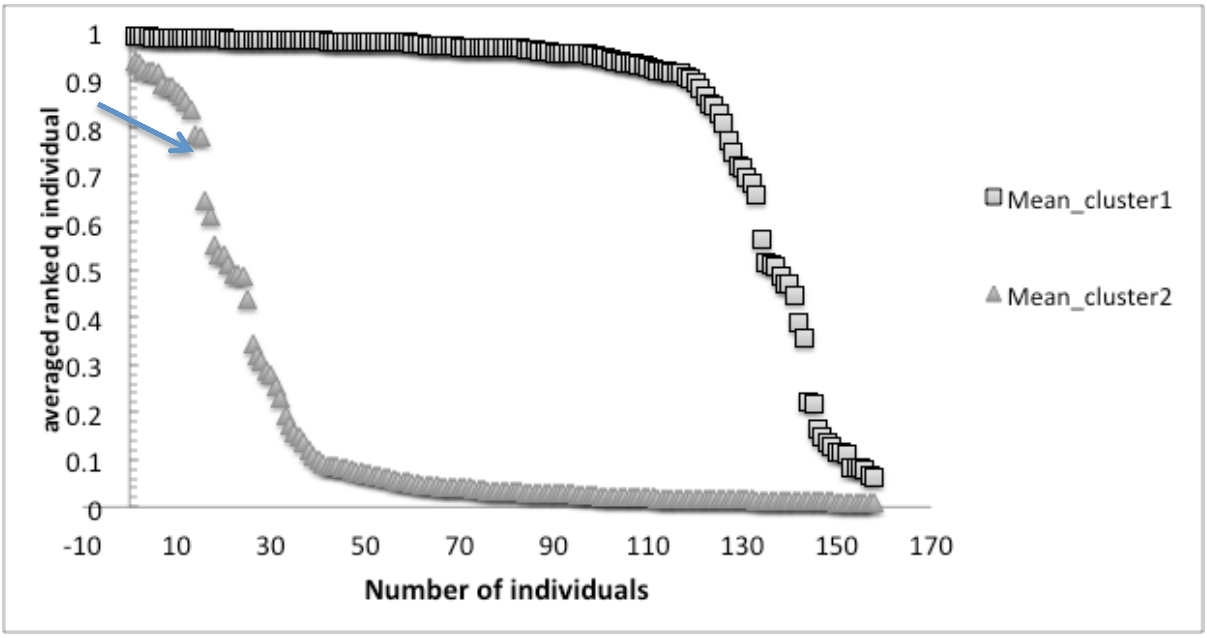

S7 Figure E Plot of ranked partial membership q of each individual to cluster 1 and 2 averaged over five independent runs. A break is present when q = 0.75 (indicated by the blue arrow).

S7 Table A Assignment of the SEN66 individuals when K = 2 per sampling site. Individuals were assigned to each cluster when the respective individual probability of assignment q averaged across the five independent runs was > 0.75 and classified as admixed when 0 < q < 0.75. In the table is indicated the number of individuals per sampling site (N), the average, the minimum (Min), and the maximum (Max) of the individual probability of assignment q, total number of males (N M) and females (N F) and proportion of Males (M %) and females (F %) assigned to each cluster.

Sampling site	N	Average	Min	Max	N M	N F	M (%)	F (%)
GD	11				6	5		
Cluster 1	9	0.96	0.9	0.98	5	4	83.3	80.0
Cluster 2	0						0.0	0.0
Admixed	2				1	1	16.7	20.0
qcluster1		0.61	0.51	0.71				
qcluster2		0.39	0.29	0.49				
SI	108				66	42		
Cluster 1	94	0.96	0.75	0.99	55	39	83.3	92.9
Cluster 2	0	0.88	0.8	0.96	0	0	0.0	0.0
Admixed	7				5	2	7.6	4.8
qcluster1		0.54	0.36	0.69				
qcluster2		0.46	0.31	0.64				
CL	11				5	6		
Cluster 1	11	0.97	0.91	0.98	5	6	100.0	100.0
Cluster 2	0	0	0	0	0	0	0.0	0.0
Admixed	0				0	0	0.0	0.0
Lk	13				5	8		
Cluster 1	13	0.97	0.92	0.98	5	8	100.0	100.0
Cluster 2	0				0	0	0.0	0.0
Admixed	0				0	0	0.0	0.0

NK	22				15	7		
Cluster 1	1	0.95			0	1	0.0	14.3
Cluster 2	11	0.88	0.80	0.95	10	1	66.7	14.3
Admixed	6				2	4	13.3	57.1
qcluster1		0.53	0.39	0.72				
qcluster2		0.45	0.28	0.61				

S7 Figure F: **Spatial autocorrelation for females and males using samples distanced at a maximum of 66 km using SEN66 and GB66.** SEN66: a - females N= 68 genotypes, n $_{\text{pairwise comparisons}}$ [0-16.5[= 1427, [16.5 - 33[= 424, [33-49.5[= 56, [49.5-66[= 371 and b - males N = 97 genotypes, n pairwise comparisons: [0-16.5[= 3041, [16.5 - 33[= 385, [33-49.5[= 75, [49.5-66[= 1155). GB66: c - females N = 74, n $_{\text{pairwise comparisons}}$ [0-16.5[= 642, [16.5 - 33[= 793, [33-49.5[= 630, [49.5-66[= 636, and d - males N = 37 genotypes, n $_{\text{pairwise comparisons}}$ [0-16.5[= 146, [16.5 - 33[= 200, [33-49.5[= 128, [49.5-66[= 192. The y-axis shows the autocorrelation coefficient *r* measuring genetic similarity (*r* > *0*) or dissimilarity (*r* < 0) between pairs of individuals separated by four distance classes (X-axis: 8.66 to 25.98 km, end point). Significant differences are indicated.

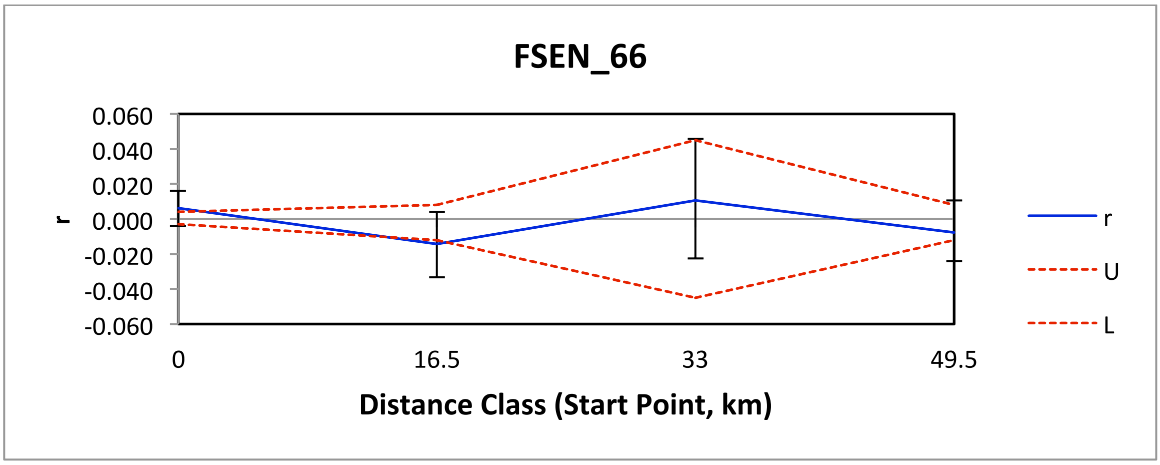

a) **Females SEN66** Significant positive autocorrelation was found at [0-16.5[(P = 0.003) and significant negative autocorrelation was found at [16.5-33[(P = 0.008).

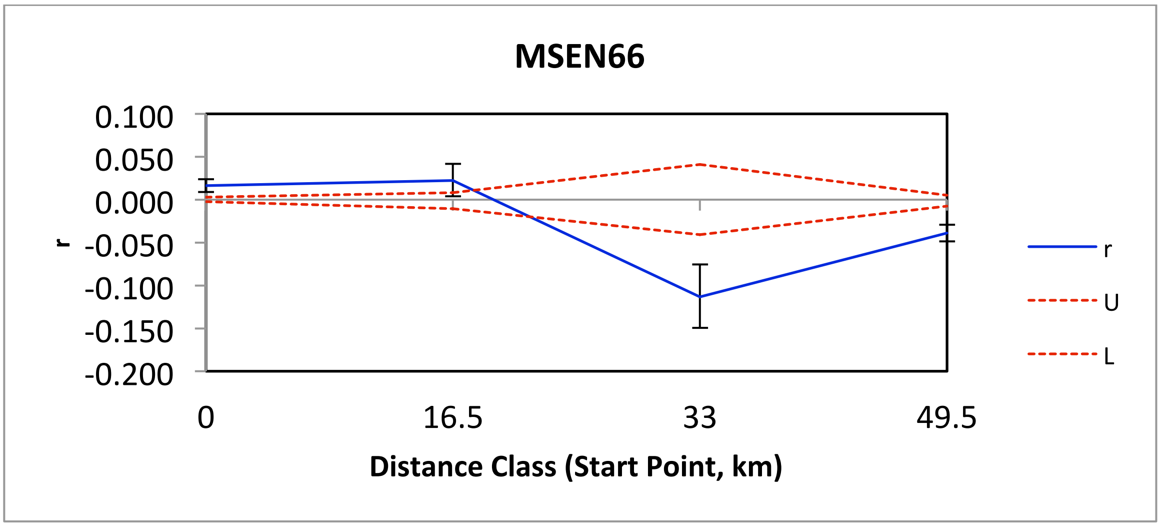

b) **Males SEN66** Significant positive autocorrelation was found at [0-16.5[(P = 0.0001) and [16.5-33[(P = 0.0001) and significant negative autocorrelation was found at [33-49.5[(P = 0.0001) and [49.5-66[(P = 0.0001).

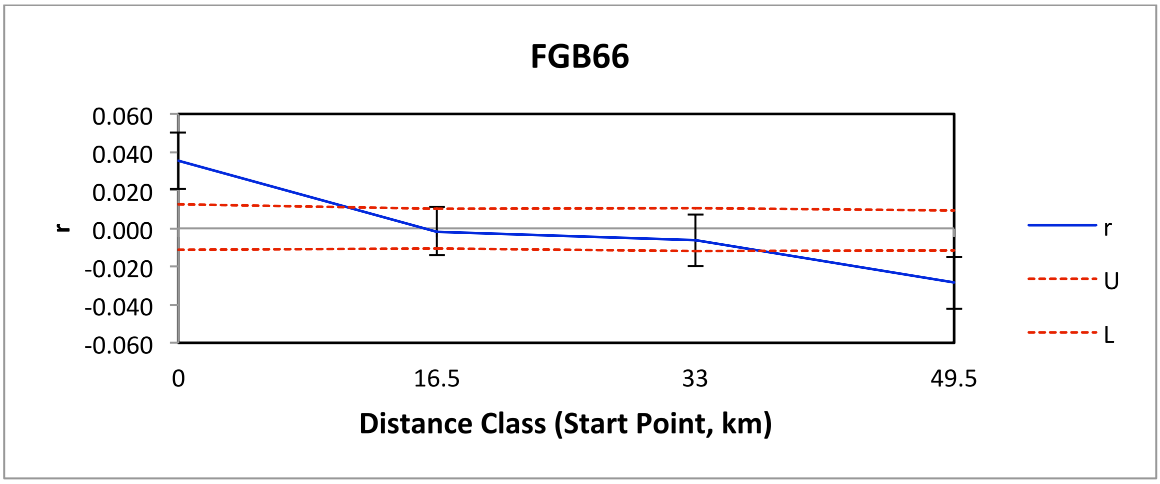

c) **Females GB66** Significant positive autocorrelation was found at [0-16.5[(P = 0.0001) and significant negative autocorrelation was found at [49.5-66[(P = 0.0001).

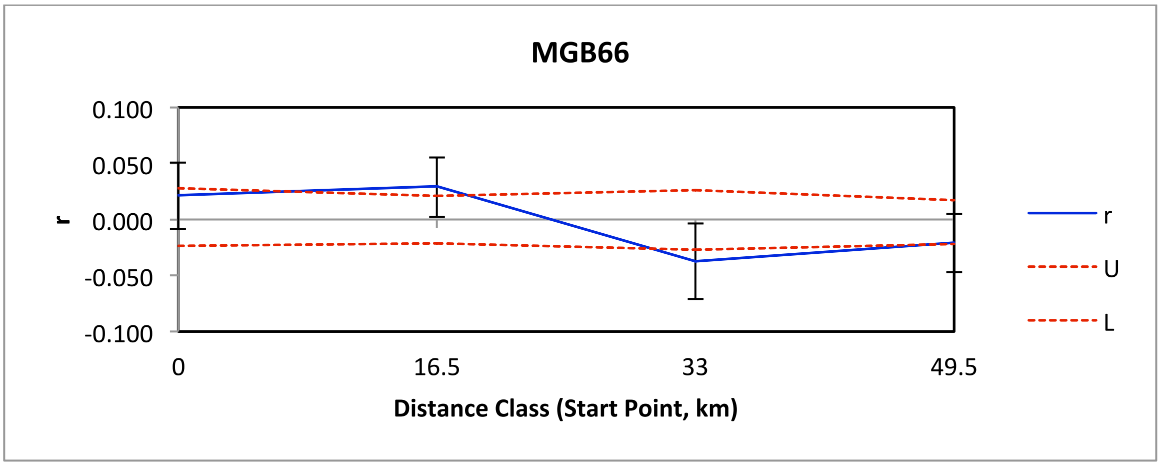

d) **Males GB66** Significant positive autocorrelation was found at [16.5-33[(P = 0.003) and significant negative autocorrelation was found at [33-49.5[(P = 0.004) and [49.5-66[(P = 0.03).

S7 Figure G Spatial autocorrelation for females and males using samples distanced at a maximum of 26 km in SEN26, GB_Cantanhez26 and GB_Cufada26. SEN26: a - females N= 61 genotypes, $n_{\text{pairwise comparisons}}$ [0-8.66[= 1376, [8.66 - 17.32[= 78, [17.32-26[= 376, and b - males N = 82 genotypes, $n_{\text{pairwise comparisons}}$ [0-8.66[= 2906, [8.66 - 17.32[= 55, [17.32-26[= 360). GB_Cufada26: c - females N = 32, $n_{\text{pairwise comparisons}}$ [0-8.66[= 171, [8.66 - 17.32[= 0, [17.32-26[= 273, and d - males N = 16 genotypes, $n_{\text{pairwise comparisons}}$ [0-8.66[= 40, [8.66 - 17.32[= 0, [17.32-26[= 78). GB_Cantanhez26: e - females N=42, $n_{\text{pairwise comparisons}}$ [0-8.66[= 287, [8.66 - 17.32[= 190, [17.32-26[= 384, and f - males N = 21, $n_{\text{pairwise comparisons}}$ [0-8.66[= 56, [8.66 - 17.32[= 57, [17.32-26[= 97). The y-axis shows the autocorrelation coefficient *r* measuring genetic similarity ($r > 0$) or dissimilarity ($r < 0$) between pairs of individuals separated by four distance classes (X-axis: 0 to 25.98 km, start point). Significant differences are indicated.

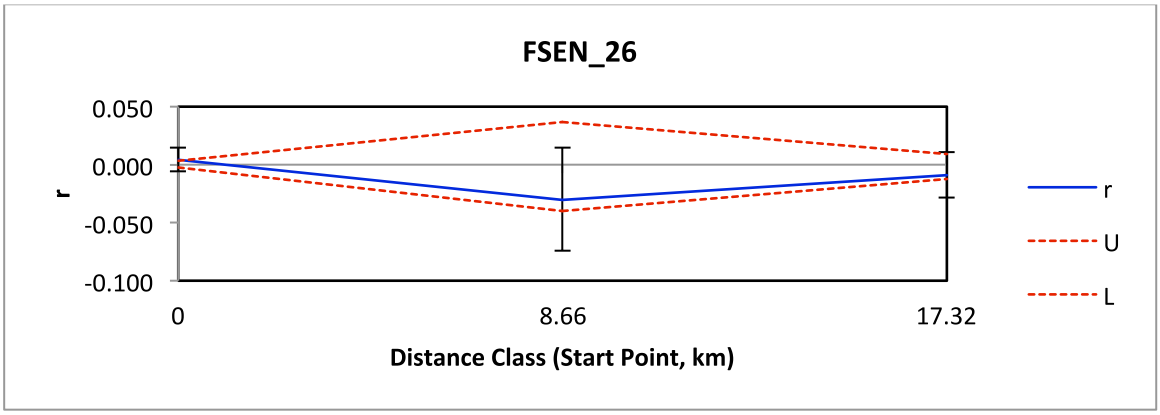

a) **Females SEN26** Significant positive autocorrelation was found at [0-8.66[(P = 0.007)

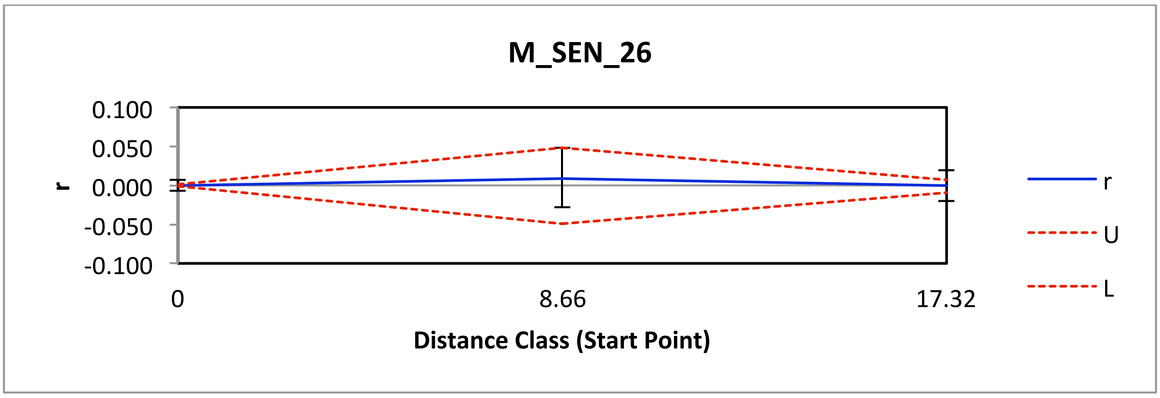

b) **Males SEN26** No significant autocorrelation was found

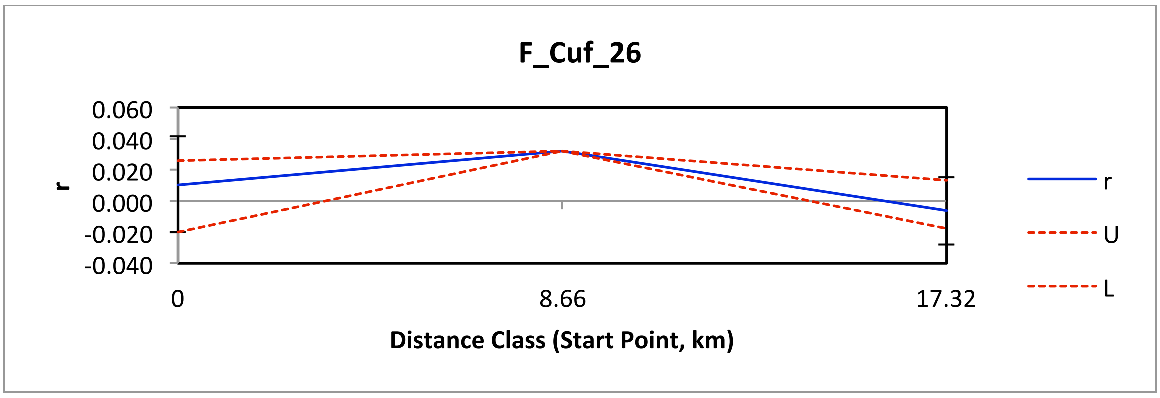

c) **Females Cufada26** No significant autocorrelation was found

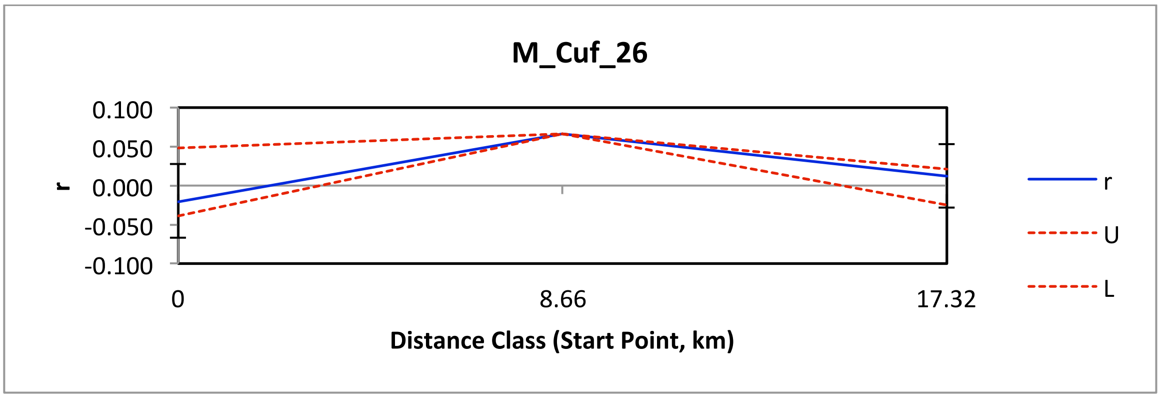

d) **Males Cufada26** No significant autocorrelation was found

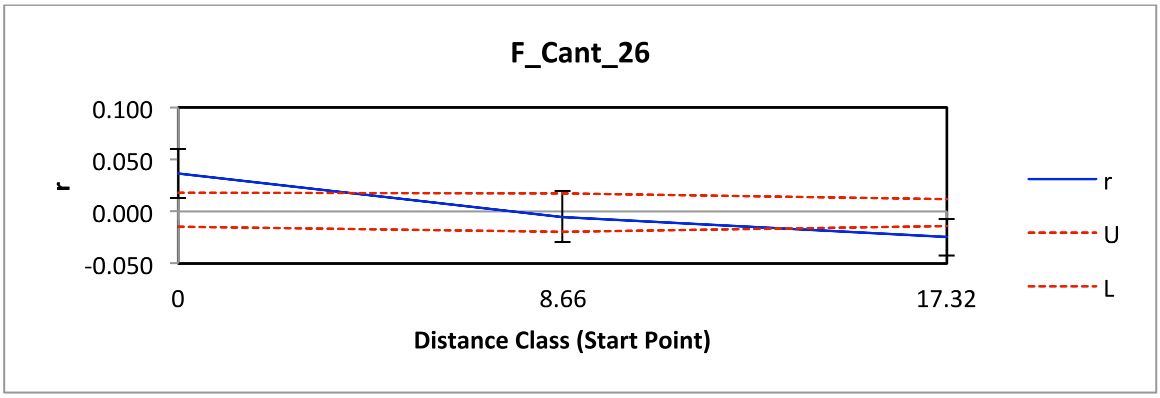

e) **Females Cantanhez26** Significant positive autocorrelation was found at [0-8.66[(P = 0.001) and significant negative autocorrelation was found at [17.32-26[km (P = 0.001)

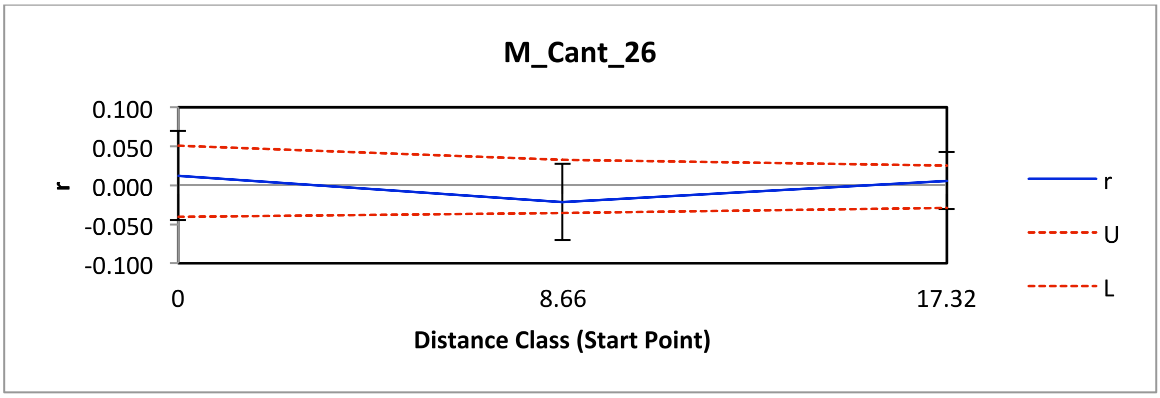

f) **Males Cantanhez26** No significant autocorrelation was found
